# Exploring the entrepreneurial landscape of university-industry collaboration on public university spin-off creation: A systematic literature review

**DOI:** 10.1016/j.heliyon.2024.e27258

**Published:** 2024-03-04

**Authors:** Alexander Romero-Sánchez, Geovanny Perdomo-Charry, Edy Lorena Burbano-Vallejo

**Affiliations:** a Unidad Central del Valle del Cauca-UCEVA, Tuluá, Colombia; b Universidad de San Buenaventura Cali, Colombia; c School of Business, CEIPA University, Medellin, Colombia

**Keywords:** Absorptive capacity, Academic entrepreneurship, Innovation in public universities, Social capital, Third mission of the university, University-industry collaboration

## Abstract

Research into the factors influencing university-industry collaboration on public university spin-offs creation has focused on management, entrepreneurship, technology and innovation. This research began with a careful systematic literature review of 4427 scientific papers published in the last ten years (2014–2023) and accessible in the prestigious Web of Science core collection. A quantitative methodology was used, complemented by the use of the visual analysis tool Posit PBC™, formerly known as R Cloud Studio. This comprehensive approach facilitated the seamless ingestion of raw data into Biblioshyni, which is a web-based platform specialised in bibliometric analysis. This review has revealed compelling trends, particularly in terms of increasing diversity, the emergence of United Kingdom as a major player, and the central role of university-industry collaboration. Our systematic review identified influential authors in the field, including the prolific contributions of scholars such as D. Radicid, S. Ropers, Y. Li and R. Owen. We also identified important research institutions, such as Utrecht University in Netherlands, Lund University in Sweden and The University of Manchester in the UK. In addition, we have shown that countries such as the United Kingdom, China and USA have made a significant contribution to the volume of publications. The results highlighted a marked increase in the phenomenon of univeristy spin-offs over the past decade, as evidenced by the exponential growth in both publication output and citation rates. This empirical revelation was underpinned by a rigorous exploration of the Web of Science database, using a carefully crafted set of keywords. Thirty-seven pivotal studies were selected for in-depth review through a sophisticated selection process that adhered to the rigorous standards of the PRISMA methodology. The aim of this review is to improve understanding and encourage deeper exploration of spin-off-based public universities through collaboration between academia and industry.

Contribution to literature

• Applying a robust quantitative methodology, complemented by the visual analysis tool Posit PBC™ (formerly R Cloud Studio), we seamlessly integrated raw data from the Web of Science core collection into Biblioshyni, a renowned web-based platform specialising in bibliometric analysis. This meticulously crafted study provides scholars with a nuanced understanding of the intricate evolutionary pathways, interconnections and nuances within the vast landscape of research topics related to public university spin-off collaboration with the industrial sector.
• The analysis of temporal dimensions and citation co-occurrences within the scientific literature offers researchers valuable insights and future research directions in the field of public university spin-offs. This perspective reveals the intricate web of connections, both temporal and thematic, that underpins the collaboration between academic institutions and the industrial sector. It also sheds light on their collective contributions to the multifaceted landscape of regional economic development.

## 1 Introduction

University spin-offs (USOs) have been recognised for their key role in improving regional and national economies, outperforming alternative technology transfer channels. They are instrumental in creating value, driving innovation, stimulating job growth and promoting overall regional economic development [1]. This phenomenon is gaining increasing attention in both academic research and practical applications, although it can be considered a specialised niche within the broader field of entrepreneurship studies [2], although it may be seen as a relatively specialised niche within the broader field of entrepreneurship studies. Emerging from university research and innovation, USOs are strategically positioned to commercialise technology and build sustainable, profitable businesses. They not only contribute financially to universities, but also serve as catalysts for job creation and significant socio-economic development. The existing literature underlines the paramount importance of supportive policies, funding mechanisms and university-industry collaborations to ensure the success and prosperity of university spin-off initiatives [3].

In recent years, universities have faced growing demands to extend their role beyond the traditional functions of education and research. The concept of “third mission” (TM) has emerged as a framework that underscores their commitment to actively contribute to society through a wide range of social, entrepreneurial and innovative initiatives. Embracing TM activities has become a key strategy for universities in today's dynamic landscape [4,5]. Universities have undergone a remarkable evolution, fundamentally reshaping their core missions of education and research. This transformation has been driven by the contemporary imperative for universities to reposition themselves as “entrepreneurial universities” [6]. Within this paradigm, innovation goes beyond mere technological advances to represent a comprehensive change in the ethos of the university, permeating all aspects of its functioning. An innovative university embodies this profound change, integrating innovation not only into its research and teaching activities, but also into its overarching vision, attitude and transformative culture. This holistic approach positions the university as a powerful catalyst for economic and social progress within its sphere of influence.

While university-industry collaborations (UIC) are promising, their successful establishment is far from assured, and in some cases these collaborations may inadvertently hinder the efficiency of technology transfer [[7], [8], [9]]. Differences in cultural norms and values between academic institutions and industry contribute to research orientations. Universities prioritise the generation and dissemination of new knowledge, often with longer development times, while industry emphasises the prompt application of existing knowledge for practical purposes [10]. In the highly competitive environment of commercial organisations, the focus is on immediate returns, low risk profiles, cost reduction and operational efficiency [11]. This stark contrast underscores the delicate balance that academia and industry must strike: Balancing the pursuit of breakthrough innovation with the need for practical, cost-effective solutions to succeed in dynamic markets [7,9].

In the course of preparing our systematic review, we came across numerous bibliometric analyses, particularly those focusing on university spin-offs (USOs) or academic spin-offs (ASOs) ([[12], [13], [14], [15], [16]]), the alternative perspective we seek to offer in this systematic literature review represents a critical step in advancing the understanding of university-industry collaboration, particularly in the area of university spin-off creation. It recognises and seeks to address the limitations identified in previous reviews, including those by Park et al. [17], Hossinger et al. [3], Abramo et al. [18], Bastos et al. [19] and Forliano et al. [20], in order to provide a more comprehensive understanding of this evolving field.

This perspective is essential, relevant and urgent for several compelling reasons. First and foremost, it is necessary to address the shortcomings of previous reviews, and it is timely given the constantly evolving academic landscape. By broadening our search parameters and introducing additional terms such as “public policies”, “regional development”, “research and development”, “absorptive capacity” and “social capital”, we broaden the horizons of our review.

Similar to previous research endeavours, our primary data source is the Web of Science database, a renowned scientific citation indexing service. This platform allows researchers to perform comprehensive citation searches across a wide range of scholarly journals. While it's worth noting that both Web of Science and Scopus are widely recognised indexing services, our preference for Web of Science stems from its focus on scholarly journals, which distinguishes it from Scopus, which covers a broader range of outlets, including general interest publications [17].

In this review, we are also attuned to emerging trends, with a strong focus on the overarching themes of the “entrepreneurial university”, “sustainability” and “developing countries”. These trends are of paramount importance in the contemporary academic landscape. They are underpinned by the dynamic evolution of entrepreneurial university models, the pressing global demand for sustainability, and the escalating influence of developing countries in research and innovation. Our perspective is not only exploratory, but also timely, in keeping with the dynamic and evolving nature of the field.

This study aims to bridge a critical gap in our understanding of university-industry collaboration, particularly in the context of university spin-off creation, and to provide actionable insights that can inform the strategic planning and execution of university spin-offs in the academic sector. We anticipate that our findings will serve as a guiding compass for navigating the complex landscape of university spin-offs, shaping future research agendas, informing policy development, and ultimately catalysing progress in this multifaceted field.

### 1.1 Research questions

Three key questions were addressed and explored in this study:

**1.1.1 Rq1.** To what extent can we distil a comprehensive understanding of the prevailing state and dynamic trajectories of UIC in the area of USOs through a meticulous decade-long examination of the annual distribution of scholarly works and their associated citations?

**1.1.2 Rq2.** In the area of UIC through public USOs creation, what are the main research interests that have received significant attention and scrutiny?

**1.1.3 Rq3.** Amidst the myriad of studies and multifaceted aspects that characterise the collaboration between academia and industry in public USOs creation, which specific ones have emerged as central and pivotal in shaping both the discourse and the progress within this research area?

To complete **Rq1**, we used a quantitative methodology complemented by the use of the visual analysis tool Posit PBC™ (formerly R Cloud Studio). This integrated approach facilitated the systematic processing and transformation of raw data into a format compatible with Biblioshyni, a specialised web-based platform for advanced bibliometric analysis. Through this methodology, we conducted a thorough examination of the annual distribution of studies and citations over the past decade, providing a comprehensive understanding of the evolving landscape of UIC in public USOs creation.

In response to **Rq2**, we identified 30 key research hotspots within the expansive field of UIC in USOs creation, covering areas such as “academic entrepreneurship”, “technology transfer offices”, “firm performance”, “innovation ecosystems” and “the third misson of the university”. Our analysis not only revealed these thematic hotspots, but also provided valuable insights into their temporal dynamics. Over the years, the prominence of these hotspots has evolved significantly. For example, between 2012 and 2017, “technology transfer offices”, “knowledge transfer”, “innovation”, “social capital” and “absorptive capacity” dominated academic discourse. However, in the more recent years from 2020 to 2023, “innovation”, “firm performance”, “entrepreneurship research” and “collaborative networks” have emerged as central areas of investigation. This nuanced understanding of the temporal shifts in research hotspots enhances our understanding of the evolving scholarly discourse and research priorities in the area of UIC in public USOs.

In response to **Rq3**, we conducted a meticulous review of the extensive academic literature on UIC in public USOs. Our exhaustive review revealed a constellation of seminal studies that have profoundly influenced the trajectory of research in the field, shaping the overarching narrative and research agenda. We also traced the evolution of key themes, including “technology transfer offices”, “knowledge transfer”, “absorptive capacity”, “the third mission of the university”, “social capital” and “entrepreneurial ecosystems”, which have emerged as critical focal points. These key themes have not only enriched the academic discourse, but have also led to transformative advances in how we understand university-industry collaboration in the context of public university spin-offs.

The remainder of this study is structured as follows: Section 2 provides a comprehensive literature review of UIC in public USOs. Section 3 outlines our data sources and analytical tools. In Section 4, we present our research findings using advanced visualisation techniques, including reference co-citation networks, landmark references, burst references, and keyword co-occurrence networks. In particular, we used Bibliometrix®, an open source R package, and VOSviewer for this analysis. Section 5 contains a detailed discussion of the findings we've uncovered, along with managerial and practical implications, accompanied by recommendations based on our findings. Finally, we conclude with a summary, address research limitations, and explore future prospects in this dynamic field.

## 2 Literature review

### 2.1 Theoretical frameworks for understanding the university agenda for creating USOs

The emerging interest in university spin-offs is based on two seminal concepts: the entrepreneurial university and academic capitalism. The entrepreneurial university, as conceptualised by Etzkowitz [21], underscores the transformation of universities from passive knowledge producers to active participants in leveraging their intellectual and human assets for economic development through knowledge commercialization and technology transfer [[21], [22], [23]]. At the same time, academic capitalism, as described by Slaughter and Leslie [24], highlights the increasingly market-oriented behaviour of universities, including investment in faculty-initiated spin-offs, reflecting their evolving role not only as knowledge generators but also as entrepreneurial entities [24,25].

The pivotal research by Miranda et al. [26] thoroughly examines the multifaceted role of universities as catalysts for academic entrepreneurship, embarking on a comprehensive exploration that spans historical and contemporary perspectives. This research delves into the complex interplay between internal and external policy dynamics. A fundamental shift in the recognition and expectation of the contribution of universities to economic development has taken place since the mid-1980s and has become embedded in regulatory systems and central to global public policy discourse. At the heart of this transformative shift is the concept of the “entrepreneurial university”, first introduced by Etzkowitz in 1983 [21].

### 2.2 The concept of entrepreneurial university

The concept of the entrepreneurial university represents a fundamental redefinition of the role of universities, moving beyond their traditional role as passive creators and disseminators of knowledge. Instead, they have become dynamic entities that actively use their intellectual and human resources to contribute to social and economic progress on a global scale [22,23]. This transformation is based on the recognition of universities as key drivers of innovation, fostering an entrepreneurial ethos that permeates all aspects of their operations, from academia to administration [1].

Universities are now recognised as primary catalysts for economic growth, knowledge diffusion and technology transfer, thereby catalysing broader social and economic progress. This redefined role reinforces the active engagement of universities in mobilising their academic and innovative resources for the common good, and affirms their potential to shape and drive socio-economic landscapes. In essence, the entrepreneurial university serves as a powerful force in steering progress and prosperity in today's world [27].

### 2.3 The university-industry collaboration

University-industry collaboration (UIC) refers to the interaction between different components of the higher education system and industry, with the primary aim of fostering the exchange of knowledge and technology in order to enhance the knowledge base of organisations [28,29]. The upsurge in collaborative activity is a direct result of converging pressures affecting both industry and academia. In the corporate domain, these pressures stem from the relentless march of technological advancement, shortened product life cycles, and heightened global competition, all of which have fundamentally reshaped the competitive landscape for firms [30].

At the same time, academic institutions are facing their own challenges, characterised by the explosive growth of new knowledge and the daunting issues of rising costs and financial constraints. As a result, universities are being forced to forge strategic alliances with business - a vital imperative to maintain their leadership in various fields of study and ensure their continued position at the forefront of knowledge generation [30,31].

In the area of university-industry collaborations, the focus is on relational dynamics, which include the extent of collaborations between universities and different companies, as well as the historical links between specific university-industry pairs. These two variables are significant in their influence on the emergence of new technology-based firms [32]. Nevertheless, it is imperative to recognise the complex interplay between the external dynamics of industry and the strategic choices made within individual universities. Together, these dynamic factors shape the intricate tapestry of spin-off creation, survival and eventual dissolution. This complex interrelationship warrants comprehensive research and understanding [33]. This knowledge gap is particularly vexing as the longevity and success of these academic spin-offs are critical measures of their overall performance. Moreover, it is a widely stated goal of the vast majority of parent universities to see their spin-offs not only survive but thrive in a competitive business environment [31].

Effective university-industry collaborations (UICs) have produced a wealth of innovation on a global scale. In this dynamic partnership, academia serves as a source of creativity, teeming with young, inventive talent, while industry focuses on innovating and advancing new products, processes and services. These innovations not only improve the quality of human life, but also generate significant financial returns [28,34]. Within this framework, participants from universities and research institutes gain a better understanding of the challenges faced by companies. The complexity of solving problems requires a joint effort, drawing on the expertise of knowledge creators and the resources provided by those with capital and means [28,35].

### 2.4 The role of public entrepreneurial universities in the university-industry collaboration

University research output, an important source of knowledge spillovers, plays a crucial role in the USOs creation [36]. Firms strategically invest in their absorptive capacity to exploit knowledge spillovers from external sources and turn this information inflow into a competitive advantage [37]. The study conducted by Davies et al. [38] focuses on benevolent knowledge sources and highlights the central role of an intellectual property rights (IPR) management initiative within the public university environment. This fresh perspective provides valuable insights into IP commercialization strategies within universities [38].

The phenomenon of university spin-offs is well established in leading research countries such as the United States, Japan, Canada, Germany and Israel, where deliberate ecosystems have nurtured these entities and overcome historical reluctance to exploit scientific knowledge from public funding sources [4]. In contrast, the university spin-off landscape in the Latin-American region is still in its infancy, with progress being made in countries such as Chile, Brazil and Colombia, but not reaching the maturity observed in more developed countries [39]. This divergence highlights the complex interplay between regional economic conditions, research infrastructure and entrepreneurial ecosystems.

In the context of promoting the role of public entrepreneurial universities in fostering university-industry collaboration, public support for start-ups commercialising technologies derived from university research is crucial. Promising technologies often face barriers in their transition to the commercial domain due to risk-averse private investors. Existing research highlights the effectiveness of commercialization-focused subsidies in facilitating favourable performance outcomes for nascent firms, supporting the potential of public intervention in fostering university-industry collaboration [40].

### 2.5 Regional economic development and social capital in public USOs fostering university-industry collaboration

Social capital encompasses the tangible and potential resources inherent in an individual's or social entity's network of relationships, including both the network and the assets accessible through it [41]. In the context of knowledge transfer, different dimensions of social capital help organisations to identify and establish valuable links with relevant partners, with university-industry collaborations serving as a context where different organisational principles can pose challenges to relationship development [42].

Different types of social capital, such as bonding, bridging and linking, specifically affect the success of UICs and USOs through the manifestation of cohesive networks such as research groups, industry consortia and collaborative projects. This form of social capital enhances collaboration and is expressed as follows.

### 2.6 Bonding social capital

Based on two fundamental pillars, i) Trust and collaboration: Strong ties foster trust between members of academic and industrial units, creating an environment conducive to collaboration. Within close-knit groups, individuals are more likely to share knowledge, resources and innovative ideas, contributing to the success of collaborative projects and subsequent spin-offs and ii) Efficient knowledge transfer: Bonding social capital streamlines the transfer of tacit knowledge within a tightly knit community. The shared understanding and familiarity between collaborators facilitates efficient knowledge sharing, reduces communication barriers and increases the overall effectiveness of collaborative initiatives [30,40,42].

### 2.7 Bridging social capital

Bridging social capital involves connections between different groups, fostering relationships between different social entities. In the context of UICs and USOs, bridging social capital contributes to: i) **Cross-sectoral collaboration**: Bridging social capital facilitates collaboration between academia and industry across organisational and sectoral boundaries. This cross-sectoral engagement brings diverse perspectives, skills and resources, enriching the collaborative process and increasing the potential for successful spin-off ventures and ii) **Diverse knowledge exchange**: Interactions across diverse networks promote the exchange of a wide range of knowledge and expertise. Bridging social capital enables the integration of insights from different disciplines and industries, fostering innovation and contributing to the creation of high-impact university spin-offs [30,40,41].

### 2.8 Linking social capital

Linking social capital involves connections with external entities such as government agencies, funding bodies and other institutions. In the context of UICs and USOs, linking social capital plays a key role in: i) **Access to resources:** Establishing links with external entities provides access to additional resources, including funding, expertise and infrastructure. Universities engaged in UIC, supported by robust linking social capital, can leverage external resources to nurture and sustain successful spin-off ventures and ii) **Policy advocacy and** support**:** Strong links with policy makers and relevant institutions increase visibility and support for UIC initiatives. Linking social capital facilitates advocacy for favourable policies, regulatory frameworks and financial incentives, creating an enabling environment for the success of university spin-offs [28,29,40].

The triple helix model, a widely recognised framework, provides insight into the complex dynamics within national innovation systems, involving university, industry and government components in different countries [43]. Its applicability extends to the study of sectoral organisational structures at the regional level. Universities have a long history of shaping regional innovation policy, with an emphasis on cultivating knowledge networks, particularly in peripheral areas. A prevailing, unwavering view suggests that every university has the potential, and sometimes the responsibility, to act as a central driver of entrepreneurship across different geographical landscapes [37]. However, marginal innovation systems face specific challenges in translating research and higher education efforts into tangible economic progress. While this challenge is not exclusive and manifests itself in different innovation contexts, it is particularly pronounced in less developed regions and nations, exacerbating disparities in business innovation, absorptive capacity and implementation [44].

## 3 Methodology

### 3.1 Data analysis and citation data retrieval

For data analysis and to obtain a comprehensive document citation dataset, this study relied on Clarivate Analytics' Web of Science Core Collection™ (WoS). Specifically, we relied on one of their renowned indexes: The Social Sciences Citation Index (SSCI). The rationale for this choice stems from the recognised inefficiency of using multiple databases simultaneously, which often leads to duplication of records [45]. Given the esteemed status of the Web of Science Core Collection™ as the gold standard database for assessing scientific performance, it was the logical and definitive choice for our research endeavours.

The first step in our research was to conduct a comprehensive search of the TOPIC field, using this database as our primary resource. This search was specifically designed to identify all publications related to collaboration between universities and industry in the context of public USOs with a publication date in the period ranged from 2014 to 2023. To ensure precision, we started our search with a query string tailored to this topic, focusing on these Web of Science categories: Management, Economics, Business, business finance, social issues. This strategic approach yielded an initial dataset of 4427 relevant documents, which formed the basis of our comprehensive analysis.

**Fig. 1:**
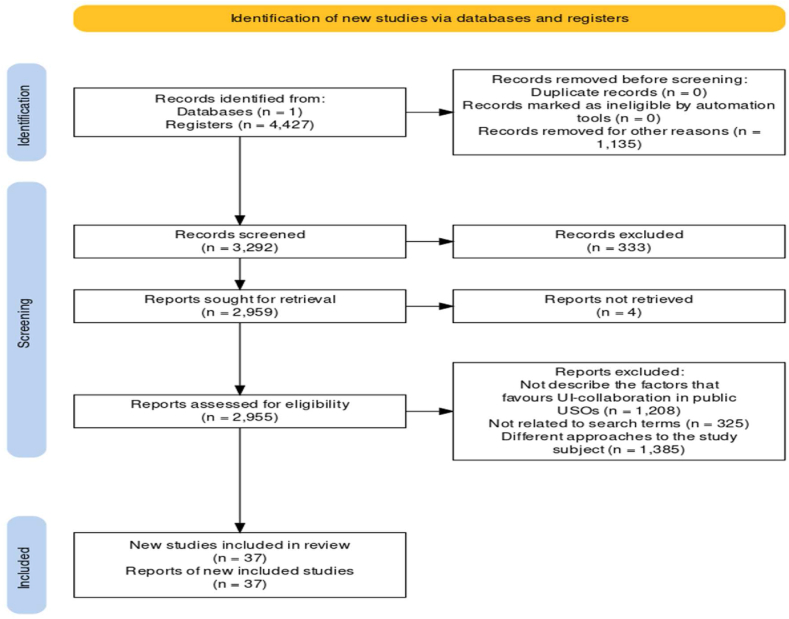
Diagram of the PRISMA statement and the steps involved in identifying bibliographic data and refining searches. Source: Modified from Haddaway et al. [47].

Following the completion of our initial search, which was originally designed specifically for systematic literature reviews [46], we made the strategic decision to incorporate the Preferred Reporting Items for Systematic Reviews and Meta-Analysis (PRISMA) statement to increase the precision of our search results (see Fig. 1). The specific criteria that guided our selection process are shown in Table 1.

**Table 1:** Criteria for the retrieval of cited documents in our data set.

Items	Criteria
Time horizon:	2014–2023
Database:	Clarivate Analytics' Web of Science Core Collection™
Citation Index:	SSCI (Social Sciences Citation Index)
The keywords combination and Booleans/Search Equation^a^:	“Academic spin off” (All Fields) OR “University spin off” (All Fields) OR “Academic entrepreneurship” (All Fields) OR “Entrepreneurial universities” (All Fields) AND “Public universities” (All Fields) OR “Entrepreneurship in public universities” (All Fields) OR “Public Universities based on spin-off” (All Fields) OR “innovation in public universities” (All Fields) AND “University-Industry collaboration” (All Fields) OR “Science-based entrepreneurial firm” (All Fields) OR “Economic impact in Academic Spin Off” (All Fields) AND “Regional development” (All Fields) OR “Research and Development” (All Fields) AND “Third mission of the university” (All Fields) OR “Absorptive capacity” (All Fields) OR “Knowledge transfer” (All Fields) AND “Public policy” (All Fields) OR “Social capital” (All Fields) OR “Innovation policy” (All Fields) OR “Public policies in entrepreneurial universities” (All Fields)
Seriation by Web of Science Categories:	Management; Business; Economics; Business finance; Social issues.
Quick filters by Web of Science:	Highly cited papers; hot papers; early access; open access; Enriched cited references
Seriation by type of document:	Only original research articles
Software used^b^:	VosViewer®; Gephi 0.10.1®; Posit PBC™ formerly known as RStudio. It is a rebranding that reflects the expansion into Python and VS Code and its web interface Biblioshiny: the shiny app for bibliometrics.
Application of artificial intelligence:	Rayyan, a web-based and mobile application designed to streamline and facilitate the process of conducting systematic reviews and syntheses of the literature.

a = The research equation shown in Table 1 does not follow any of those outlined by other authors. Therefore, the study is a robust template for possible replication, for which it is sufficient to follow the research and eligibility criteria used here.

b = Adapted from Aria and Cuccurullo [49].

Three key considerations underpinned the adoption of the PRISMA statement as our guiding framework in our unique context: *i*) Due to its comprehensive nature, it has an esteemed reputation within the academic community [50]; *ii*) the growing trend of its use in a wide range of recent bibliometric studies [47] and *iii*) the inherent potential of this statement to enhance the reliability and consistency of our verification process across a number of dimensions is significant.

We chose Rayyan to complement the PRISMA Flow Diagram and enhance the systematic review process by introducing efficiency, automation and collaboration capabilities. While the PRISMA flow diagram remains essential for transparent reporting of the review methodology and results (Fig. 1), Rayyan's digital platform optimises the execution of the review. It accelerates the screening phase, reduces the likelihood of bias through customisable screening criteria, and promotes collaborative research through real-time tracking and seamless integration with reference management tools such as Mendeley, which was used in this study.

The toolkit used in this study, which is readily available online, includes the R package and has a number of powerful features: *i*) It allows users to easily generate systematic review flow diaghrams that comply with the most recent updates of the PRISMA statement [46]; *ii*) it facilitates the adaptation of GitHub code to create and distribute a user-friendly, web-based tool (a Shiny app). Remarkably, this tool enables researchers without any previous coding experience to generate publication-quality flow diagrams [50] and *iii*) it also enables users to create interactive versions of these flow diagrams, complete with hyperlinks to specific web pages, files or document sections [50] (see Fig. 1).

In order to illustrate the pragmatic utility of the PRISMA protocol in the field of management science and specifically in subjects related to the aim of this study, UI collaboration and USOs, a selection of recent studies deserve attention. Notable among these are the works of Wegner et al. [51], Padilla Bejarano et al. [52], García-Lillo et al. [53], Fauzi [54] and Pertuz et al. [55]. These reviews stand out as compelling examples that meticulously adhered to the rigorous PRISMA guidelines throughout the phases of identifying, selecting and critically appraising the literature, as visually illustrated in Fig. 1.

Our systematic review adheres carefully to the rigorous research protocol introduced by Donthu et al. [56] and closely follows the bibliometric approach outlined by Bastos et al. [19]. This method is underpinned by a qualitative framework that facilitates in-depth exploration of the corpus of published research, covering a wide range of dimensions, including citations, co-citations, authorship, co-authorship, bibliographic coupling, keywords and journal affiliation. By applying this comprehensive analytical framework, we uncover a nuanced understanding of the scientific landscape and the intricate web of knowledge diffusion in the field of university spin-off creation through UIC. This approach ensures a robust and well-informed basis for our review, thereby enhancing the quality and reliability of our findings.

In addition to considering various alternative approaches, we introduced a pioneering methodology by incorporating Bibliometrix® - an open-source R package thoughtfully designed by Aria and Cuccurullo [49]. This powerful tool is specifically tailored for quantitative research in bibliometrics and scientometrics, and includes a comprehensive set of essential bibliometric analysis techniques. Impressively, Bibliometrix® seamlessly interfaces with major databases such as Clarivate Analytics' Web of Science, Scopus, Digital Science Dimensions, PubMed and Cochrane. In addition, this versatile software offers the flexibility to export analyses in a variety of file formats, including. bibtext and. xlsx, making it an invaluable tool for researchers. As shown in Table 1, Bibliometrix® produces insightful outputs that contribute significantly to our analytical capabilities.

### 3.2 Eligibility criteria of the included studies

In the context of this research, our search for relevant studies related to public USOs involved a meticulous approach involving the use and merging of a comprehensive set of search terms. These terms included “university spin-off” and “university-industry collaboration” in their various iterations and permutations. Our primary focus was on studies delineated by the following Web of Science categories: Management; Business; Economics; Corporate Finance and Social Issues, in line with the thematic scope of our research (see Table 1). To ensure the highest level of rigour and quality, we further refined our search by imposing specific publication date restrictions. Only articles published in rigorously indexed scientific journals using a rigorous double peer review system were included in our review, thereby reducing the inclusion of lower quality, non-refereed studies.

### 3.3 Refined selection criteria

Our selection criteria were carefully designed to ensure the inclusion of articles in which academic spin-offs were the central focus of the investigation. Conversely, studies that only mentioned academic spin-offs in passing, but focused on alternative transfer processes such as collaborative agreements with external firms or exclusive patent licensing arrangements, were deliberately excluded from our analysis. Exclusions were also made for the following reasons: *i*) articles that were tangential or deviated from the core focus of our research, which is on academic spin-offs in the specific context of public universities; *ii*) studies that dealt with parameters and aspects beyond the scope of our present study. For further clarity, please refer to Fig. 1 and Table 1.

In order to ensure the veracity and integrity of the dataset, a meticulous process has been adopted [47] whereby raw files have been exported to the Biblioshiny web interface in “BibTex” format. This careful approach ensures that the data remains in its unadulterated form, ready for rigorous analysis and scholarly scrutiny. This rigorous methodology and comprehensive dataset [46,47] together contribute to a deeper understanding of the intricate nuances within the realm of academic research.

As part of our research enquiry, we began by collecting and systematically analysing bibliographic data from peer-reviewed journal articles. This valuable data set, formatted in plain text (.txt), was subjected to meticulous scrutiny using Gephi 0.10.1®, a versatile network analysis tool, which took on the role of data processor. This advanced rendering engine was used to create visually expressive and insightful representations of complex networks, facilitating a deeper understanding of the intricate structures and dynamics that underlie them [57].

In the final phase of our methodological design, we meticulously classified each article according to its specific research approach, as visually detailed in Fig. 1. This classification marked the first stage of our extensive content analysis of the original research articles that were the foundational basis of our study. This multifaceted content analysis not only helped to categorize the articles according to their overarching research objectives, but also enabled us to extract broad insights and formulate overarching conclusions regarding their primary findings. These synthesised findings, in turn, serve as the cornerstone for the subsequent section of this systematic review, providing an important bridge for in-depth exploration and discussion.

While bibliometrics provide insights into publication patterns in major databases, content analysis serves as the linchpin for constructing the conceptual framework [58]. Our study carefully relies on a carefully curated selection of 37 original research articles, visually presented in Fig. 1 and comprehensively detailed in Table 2, Table 3.

**Table 2:** Number of publications by type of data and research methods applied on 37 foundational studies on USOs creation through UIC.

Research methods	Number of publications
Quantitative –regression models	4
Mixed (Quali-Quantitative)	11
Quantitative –descriptive	7
Quantitative –other	3
Qualitative –case	6
Qualitative –interviews	2
Qualitative –comparative analysis	3
Network analysis	1

**Table 3:** Type of data, methodological approaches and research limitations across 37 foundational studies of USOs creation through UIC.

Research approach	Type of data	Research Methods			Research Limitations	Reference
		Qualitative	Quantitative	Mixed		
To identify the relevant schemes and structures that shape the UVC archetypes of 11 European UVC funds as a source of early stage financing for USOs	Qualitative data through interviews and archival data were collected for this study	Using a qualitative case study methodology, this study conducts an inductive cross-case analysis of 11 European UVC funds through the lens of an archetypal approach to institutional theory			The limitations of the research are that it is based on informants' accounts and recollections, the analysis covers a limited sample of cases, and the archetypes identified do not provide a complete picture of the UVC landscape.	[59]
To discuss academic spin-offs (ASOs) as an expression of the value of university investment in technology transfer (TT)	The type of data used in the statistical analysis were the economic variables and the parameters of the IC	The study adopted 4 methodologies to address all value drivers: discounted cash flow (DCF), the income approach from an accounting perspective, the multiples approach and the venture capital approach to determine the market value of companies.			The study has a small sample size, which has its limitations from a statistical point of view, even though it represents 70 per cent of the population	[60]
The research approach involved an in-depth evaluation of public documents from the University of Campinas, followed by personal interviews with 4 categories of actors within the organisation and an additional interview with a large multinational company.	The type of data analyzed in this study were interviews	The research methods used in this study included an in-depth evaluation of public documents from the University of Campinas and personal interviews with 4 categories of actors within the organisation, as well as an additional interview with a large multinational company.			The study has several limitations, including access to longitudinal information and the theoretical complexity of the phenomenon. The collection of longitudinal information should be considered in future research. The social impact/effectiveness of university/regional capabilities for frugal innovation should also be measured in a future study.	[61]
The research approach for this paper is quantitative and empirical, using cross-sectional data analysis to investigate the impact of both horizontal and vertical team diversity on the performance of academic spin-off firms.	Unique dataset of Italian ASOs developed by the Center for Innovation and Entrepreneurship at the Università Politecnica delle Marche and Scuola Sant’Anna in collaboration with Netval (the Italian association of technology transfer offices in universities and other public research institutions). The dataset includes the whole population of Italian ASOs for the period 2000–2007 (N = 290).	The study uses quantitative data analysis techniques to examine the relationships between team diversity and firm performance, this study tested hyphotesis and estimated two models, both Poisson regression models based on the number of patents and licences and adjusted for spin-off age.			The study has several limitations, including: investigating ASOs only in the 1st years of their creation, focusing on the power of academics versus non-academics without considering the diversity within the academic subgroup, and focusing on a single country (Italy).	[62]
The research approach used in this paper is empirical research.	The authors collected data from a sample of biopharmaceutical firms in China and used statistical analysis to test their hypotheses on the relationship between university-industry collaboration and firm innovation.	The authors used empirical research methods to test their hypotheses.			One limitation is that the study only focuses on the biopharmaceutical industry in China, so the results may not be generalizable to other industries or countries. Another limitation is that the study only examines the impact of university-industry collaboration on firm innovation, and does not consider other potential outcomes such as knowledge spillovers or social welfare. Finally, the study relies on self-reported data from firms, which may be subject to bias or measurement error.	[63]
The aim was to show descriptive data about the researchers' characteristics, organizational factors, and university-industry collaboration, and to analyze the differences in UIC based on the researchers' gender, age, and seniority using ANOVA	The data used in this study was collected through a survey that was sent by email to the researchers affiliated with a public university in Mexico and participating in university-industry projects. The data collection was carried out from March to May 2016, and the entire sample of 177 researchers responded to the survey.	The research methods used in this study was descriptive, exploratory, and quantitative			The study only focuses on researchers affiliated with a public university in Mexico, and the findings may not be generalizable to other contexts and other factors such as academic discipline, research funding, and institutional policies, may also play a role.	[64]
The approach involves 2 evaluation stages consisting of policy analysis and learning evaluation based on learning system modelling	This study employed a quantitative approach to explore the questionnaire data through several stages. The data was collected through surveys of both internal and external stakeholders, including students, alumni, and industry representatives. The study analyzed the data using statistical methods such as normality tests, correlation analysis, and hypothesis testing based on one-tailed student t distribution.	The research methodology used in this study was a quantitative approach			It should be recognised that this research serves as a call for reform of the national innovation system. Such a reform should include giving a greater role to the university-industry partnership, which contributes significantly to technological progress. However, it is important to recognise that the capacity of the study to prescribe specific reform strategies or to assess the practical feasibility of this proposal is limited.	[65]
The research approach used in this study was a mixed methods approach	The type of data analyzed in the study was qualitative data	The research method used in this study was a 3-stage mixed methods approach. In the 1st stage, case studies were conducted through interviews with HEIs and their industrial partners. In the 2nd stage, negative binomial regression models were estimated on data gathered from the websites of 2280 Indian HEIs. In the third stage, the impact of collaborations on graduates' employability competencies.			The data collected on teaching collaborations and their determinants from websites are constrained by the ‘controlled’ nature of the information available on the website and the possibility of human error.	[66]
The research approach involves conducting a survey to gather data on university-industry cooperation in a low innovative region, comparing low tech and high-tech clusters, and utilizing statistical analysis (Chi-square test) to identify significant differences in motivations, channels, and barriers to cooperation.	The type of data used in the study was survey data and information collected through open ended interviews	The research methods used in this study involved analyzing the cases of 4 clusters from both high tech and low-tech industries in the Region of Campania (southern Italy). The study collected data through an online survey and also used data from semi structured and face to face interviews with the clusters' presidents			The sample size could be expanded by increasing the respondent rate and considering more clusters within the Region of Campania to conduct a deeper exploration of the variety of industry effects on patterns of UIC.	[67]
The primary aim of the study is to analyze and understand the nature of entrepreneurial activities in the creative arts and humanities, focusing on the benefits and beneficiaries of such activities.	The type of data used in addition to the survey was institutional data provided by the “Higher Education -Business and Community Interaction Survey 2007–08″, which includes questions on 1/3 stream activities and funding.	The research methodology used in this study is quantitative. The study used a combination of survey data and regression analysis to investigate academic entrepreneurship in the UK higher education sector and views on academic entrepreneurship.			Limitations including the fact that some activities common in the creative arts and humanities were not included in the survey, and there is limited information on the nature of benefits and beneficiaries by type of activity.	[68]
The research approach in this document is exploratory in nature. The authors analyze the structural aspects of public research and business innovation systems to understand the design and efficiency of policies for technology transfer (TT) and research commercialization. They also explore how national policy mixes have adapted to these structural aspects.	The type of data used in this paper is the European Commission/OECD Science, Technology and Innovation Policy (STIP) Database	The research method adopted is mixed. The data sources and methodology used in this study focused on operational definitions and policy initiatives, accounting for all policy initiatives on the same scale.			Overlooking the magnitude of policy intervention in terms of budgets. Additionally, the study highlighted the lack of appropriate and comparable data for budget-based quantification of policies.	[69]
The research approach in this study involved analyzing the impact of government subsidy strategies on university-industry collaboration and sustainable innovation. The authors developed a three-stage Stackelberg game model to examine the relationship between government subsidy rates and the profit generated by the collaboration.	The government subsidises databases for universities and research institutes.	The research method adopted in this study is mixed			The model does not incorporate institutional and relational factors which play a crucial role in industry university collaborations, neither distinguish between large companies and SMEs, although their motivations to collaborate in R&D with external partners are different and the leverage effect of R&D subsidies for SMEs is evident	[70]
The study assesses the impact on business innovation and academic scientific production, analyzing structured data to investigate the effects of university-industry relationships in a specific regional context with low absorptive capacity.	The type of data is a structured data set containing information on researcher general characteristics, formal linkage activities with external agents, research activities financed by public funds, and scientific production (ISI indexed publications)	The study employs a combination of quantitative research methods, using structured data, to analyze the effects of university-industry relationships. Qualitative research methods may have been used to gather additional insights from participants or experts in the field			Is dangerous to generalize based on findings from a single case study, and therefore, it would be valuable to replicate this research in other contexts and the dependent variables used to analyze the impact of UIRs on firms' innovation and scientific production may not take sufficient account of the spectrum of possible results	[71]
The research approach used in this study was a deductive approach. The data collected included information about the importance of university-government research collaboration, the generation and transfer of new ideas, and the production and commercialization of new knowledge for industrial use in China.	In this study, the researchers used qualitative data collected through semi-structured focus group interviews with university team leaders and team members, as well as interviews with anonymous government experts.	The research method adopted in this study is qualitative			The researchers selected only the top three Hefei city-based public sector universities and anonymous government experts from Anhui province of China. The selected interviewees were fluent in English, although they sometimes needed help from a translator or language translation software tools to translate and explain questions using the Chinese language.	[72]
The research approach used in this article was a qualitative, multiple case study approach based on three university-industry partnerships involving DSM, the multinational corporation that has more than 40 active university-industry partnerships with universities, other research institutions, and several companies of various sizes	The text is a qualitative, multiple case study approach combining qualitative methods—interviews, supplemented by document analysis—and using open-ended questions.	The research method adopted in this study is qualitative			A more diverse data collection approach, such as observational methods or experimental designs, could enhance the robustness of the findings. Longitudinal studies or extended data collection periods could provide a more nuanced understanding of the phenomenon.	[73]
The research approach used in this study was a qualitative survey of Italian academic departments, which provides valuable insights into the perceptions and experiences of academic researchers	Type of data used in this study was a qualitative survey of Italian academic departments. The authors conducted interviews with 197 university departments in Italy to gather data on the main obstacles to technology transfer activity as perceived by academic researchers and their impact on university-industry collaborations	The research method adopted in this study is qualitative			In an Italian academic context, the results of this study may not be generalizable to other countries or settings due to potential differences in barriers to collaboration and technology transfer. In addition, the reliance on self-reported data from academic researchers may introduce response bias and inaccuracies, as individual circumstances and biases may influence their perceptions. Furthermore, the study did not explore the perspectives of industry partners and other stakeholders, which could provide a more comprehensive view of barriers to collaboration.	[74]
The research approach used in the study involved comparing selected indicators for European countries. The study analyzed data from 2465 higher education institutions from 36 European countries. Regression models, including probit and logit regression techniques, were used to compare and validate the results.	The study utilized secondary data from the European Tertiary Education Register. The database contained data for over 2400 institutions from 36 European countries. The data were available for the academic years 2011/2012, 2012/2013, 2013/2014, and 2014/2015.	The research methods applied in this study were primarily quantitative. The study utilized regression analysis, specifically probit and logit regression techniques, to examine the potential factors affecting the establishment of university spin-offs. The analysis involved comparing and validating the results using different regression models.			The creation of university spin-offs is a unique event, and the data only captured spin-offs created in the 2011–2014 period. It is possible that more spin-offs were created in the past, but were not captured in the data. The study focused on European countries, and the findings may not be generalizable to other regions or countries outside of Europe. The research approach was primarily quantitative, and other qualitative methods, such as interviews or case studies, were not utilized. This limited the depth of understanding and potential insights that could have been gained from qualitative data.	[75]
The research approach was an online survey translated into 22 languages and undertaken in 33 countries in Europe and the European Economic Area	The type of data used in this study was an online questionnaire	The research method adopted in this study is qualitative			Since the data used in this study was collected in the framework of a European project, there was no common measure to gauge the impacts of ‘‘barriers’’ and ‘‘drivers’’ on ‘‘academic entrepreneurship’’. The dependent variable in this study, ‘‘extent of academic entrepreneurship’’, was self-reported; further research could concentrate on developing a more objective measure of academic entrepreneurship	[76]
This study adopts an institutional approach to investigate and explain the processes underlying the establishment and development of partnerships between academic institutions (universities/faculties) and industry stakeholders.	The paper uses a unique dataset comprising 1158 contracts with industry at the 25 faculties belonging to five technical universities in Slovakia. Negative binomial regression analysis is used to evaluate the determinants for academic engagement in contract research.	The research method adopted in this study is mixed.			The outcomes only concern technical universities in Slovakia, and there is still room for analysis of other faculties comprising other subject areas. Since there is no longer time series data, the 2014–2016 timeframe did not permit to explore additional contexts in the model.	[77]
The research approach in this study adopted a mixed methods strategy, combining qualitative and network data collection methods. Qualitative data were collected through face-to-face or telephone interviews with academic entrepreneurs, using an open-ended interview template. In addition, network data was collected using a name generator technique to identify key contacts who were critical.	The type of data used in this study was a combination of qualitative data from interviews and quantitative data from network data collection. The interviews provided qualitative information about the experiences and perspectives of academic entrepreneurs, while the network data collection involved gathering information about the contacts and connections of the entrepreneurs.	The research method adopted in this study is qualitative.			The study has several notable limitations. First, the sample size is notably small and limited to a single US state, which limits the broader applicability of the findings. Second, the use of static data collection methods may not fully capture the dynamic nature of network dynamics and their potential influence on spin-off success. Third, the study's focus on academic entrepreneurs may inadvertently exclude the experiences and perspectives of entrepreneurs from diverse backgrounds. In addition, the study's reliance on self-reported interview data introduces the potential for inherent biases and inaccuracies.	[78]
The research approach used in the study exploring the interactions between firms in Andalusia and universities was an exploratory strategy based on empirical observations.	The study utilized a government-produced firm directory designed to assess innovation. This directory, created to enhance firms' engagement with regional innovation programs and other businesses, provided data on a diverse set of firms in Andalusia. The survey questionnaire was then administered to 737 innovative firms from various sectors, sizes, and innovation profiles within the region.	The study employed a mixed research methodology to investigate firm-university interactions in Andalusia. This approach involved a survey with 737 innovative firms, factor analysis to reveal relationship patterns, cluster analysis to categorize firms by their university interactions and econometric analysis to assess determinants of various interaction mechanisms.			the research acknowledges the diversity of contexts in which university-industry interactions occur, potentially limiting the generalizability of its findings. Secondly, variances in measurement instruments and operational procedures across different studies pose challenges in comparing results. Thirdly, the utilization of general innovation surveys may not adequately capture the full spectrum and intensity of university-industry connections, as firms may identify specific activities in their interactions not typically addressed in these surveys. Furthermore, common online surveys employed in this research often offer limited depth and complexity in their questions. Fifth, a tendency to treat university-industry interactions as a homogeneous entity rather than differentiating between various forms may obscure the specific dynamics and nuances of collaboration. Lastly, the operational procedures utilized in the study, encompassing online surveys and face-to-face interviews, carry their inherent limitations, affecting the range and depth of questions and information that can be obtained.	[44]
The research approach used in this study was a multilevel approach	The text presents empirical data to support the argument	The occurrence of simultaneous discoveries has been exploited to address the issue of qualitative differences between research projects with and without industry collaborators, as the research method used in this study is qualitative.			The limitations of the study are: measuring knowledge spillovers by focusing on knowledge provided by the public sector does not capture the total amount of potential local knowledge flows; private sector research activities generate knowledge spillovers that should ideally also be considered; the multilevel approach is based on a rather small unbalanced panel, which is not ideal as larger sample sizes could substantiate the recommendations derived from the results; and further control variables could be included in the analyses.	[36]
The research approach used in this article was based on theoretical sampling, where the analysis of two case studies was expected to fertilize and substantiate the understanding of the five models of third mission activities	The authors used a combination of primary and secondary data. Primary data was collected through interviews and a workshop with key stakeholders involved in third mission activities at two Danish universities. Secondary data was collected through the analysis of websites, job advertisements, annual reports, PowerPoints, evaluation reports and national documents related to drone research.	The study used qualitative research methods, specifically interviews and a workshop, to collect primary data. The data was then analyzed using qualitative data analysis techniques, including deductive and inductive coding, to identify themes and patterns in the data.			One limitation is that the study focuses on only two Danish universities, which may limit the generalizability of the findings to other universities or contexts. Another limitation is that the study relies on self-reported data from respondents, which may be subject to social desirability bias or other forms of response bias. In addition, the study only focuses on third mission activities related to drones, which may limit the applicability of the findings to other sectors or industries.	[79]
This study adopted a cross-sectional approach.	The authors collected data through a survey of 1008 UICs in the US, Japan and South Korea and used structural equation modelling to analyze the data. They also used various statistical and methodological tools to control for common method variance.	The research method was quantitative and involved the analysis of survey data using statistical techniques.			The data were collected from the perspective of the company and did not take into consideration the perceptions of the university partners. Second, the study is cross-sectional, which limits its ability to capture the relational dynamics of UICs. Thirdly, the study did not consider potentially competing explanations and reverse causality cannot be strictly ruled out. Fourth, the study was limited to the US, Japan and South Korea, and additional research in other regions and emerging economies is needed to further advance our understanding in this field.	[80]
This study adopted a qualitative approach, using thematic analysis to interpret the data. This data analysis strategy allows complex data to be understood, through the development of categories and themes summarised in the raw data. This is particularly useful for understanding processes rather than outcomes.	Type of data used in this study was qualitative, employing purposive and snowball sampling to identify experts. The semi structured interview was chosen for information collection, and a thematic analysis was employed for data interpretations	The research method adopted in this study is qualitative			This study took place in Colombia, one of the five most important emerging economies in Latin America. Firstly, it addresses institutional factors that influence university conditions for spin-off creation. However, other aspects, such as the social context, the individual characteristics and motivations of entrepreneurs, and the existence of technology transfer offices and sponsored research should be considered as determinants of spin-off creation. It is therefore important to continue research on these aspects. Second, the study used a purposive sample, an aspect that introduces community bias, due to an overemphasis on social network cohesion	[81]
The research approach used in the study mentioned in the document was a combination of quantitative and qualitative methods.	The type of data used in this study was interviews with academic inventors of several patents in force, owned by Portuguese public universities	The research method adopted in this study is mixed			The main limitation is the limited sample size of academic inventors. In addition, the study suggests that future data collection should also take into consideration the experiences of academic researchers who choose not to patent.	[82]
The research approach used in this empirical study was a 3SLS (Three-Stage Least Squares) estimation method. This method was applied to a sample of 137 research groups from the years 2006–2010. The study analyzed various variables to examine the relationship between university-industry relations and research group production.	The type of data used in this study was 137 research groups databases from Spanish universities, total number of articles published by all group members in journals included in WoS	The research method adopted in this study is quantitative.			The study only includes research groups with at least 4 years since their creation, which may introduce bias as newer groups are excluded. This could limit the generalizability of the findings to all research groups. The information regarding scientific production and university-industry relations is obtained from a research group's productivity report. This reliance on self-reported data may introduce bias or inaccuracies in the measurements.	[83]
The research approach in this study is primarily quantitative, using survey data from various sources, including the Norwegian CIS and register data from Statistics Norway to measure firm characteristics. In addition, information from Scopus is used to measure the characteristics and, more importantly, the research intensity of Norwegian universities.	The type of data used in this study was survey data from various sources. Firm characteristics are measured with data from the Norwegian CIS, supplemented with register data from Statistics Norway.	The research method adopted in this study is mixed.			The limitations notwithstanding, this research raises a word of caution about the role of research intensity at universities for creating partnerships within the local environment and, therefore, for innovation activity and growth. The study is limited to R&D collaboration and is not able to identify other ways in which firms interact with universities.	[84]
The research approach in this study adopted a case study methods strategy.	The study used qualitative data obtained from several semi-structured interviews to analyze the barriers to university-industry cooperation in the academic region III of Angola.	The research method adopted in this study is qualitative			The study focused only on the Academic Region III of Angola, which includes the provinces of Cabinda and Zaire. The findings may not be applicable to other regions or countries. The research involved only six interviews with representatives from universities and industries. The small sample size limits the generalizability of the findings to a larger population	[85]
The research approach used in this study was a qualitative survey of Italian academic departments, which provides valuable insights into the perceptions and experiences of academic researchers	Type of data used in this study was longitudinal, multi source survey data	The authors employed a mixed method research design to develop a comprehensive understanding of the phenomenon through unique insights gained from different research methods. They conducted semi structured interviews with research scientists and senior managers, developed a large scale multi source survey, and asked senior managers at DrugCo to review the performance of eligible projects			The limitations of this study call for future research. We should note that the boundary condition of the findings concerns both the type of collaboration and the task environment in which collaboration teams are embedded and this findings may not be readily applied to purely transactional university-industry collaborations (e.g., contract research—D’Este and Patel, 2007; knowledge transfer—Agrawal and Henderson, 2002; Bekkers and Freitas, 2008) or teams working on repetitive, highly routinised tasks. Future research is needed to validate these findings across various collaboration projects of different degrees of complexity and uncertainty.	[86]
The research approach used in the study is a conceptual framework based on the stakeholder theory. The study applies a holistic approach, considering stakeholders at different levels of analysis, including individual-level engagement (university staff and students) and organizational-level engagement (collaboration with academic-based business units).	The study utilized data from the Higher Education Business and Community Interaction Survey, which is collected by the Higher Education Statistic Agency. The data includes information on university income, independent explanatory variables, exogenous control variables, and interactions between stakeholders. The dataset covers a sample of 139 UK universities over a period of seven years (2010–2016).	The study employed quantitative research methods, including regression analysis and statistical tests, to examine the relationship between stakeholder involvement and university income generation.			The study focused on a sample of 139 UK universities over a specific time period (2010–2016). The findings may not be directly applicable to universities in other countries or different time periods. The generalizability of the results to a broader context should be approached with caution. The study relied on data from the Higher Education Business and Community Interaction Survey, which may have limitations in terms of accuracy and completeness. The availability of certain variables or the quality of data may vary across universities, potentially affecting the robustness of the findings.	[87]
The research approach used in this study is the extended case method combined with longitudinal case study design	The type of data was narrative accounts and factual descriptions	The research method adopted in this study is mixed			Future studies should perform more detailed investigations of selected collaborations, preferably by collecting data in real time to more closely represent the actual events. Clearly, there is a need to better understand the social capital mechanisms underlying inter-organizational collaboration and the dynamics of these mechanisms over time. Relying exclusively on qualitative studies of social capital may be overly descriptive, however, which suggests the need for mixed methods to obtain a more comprehensive understanding of how networks are generated and of the process linkages among different social capital dimensions	[42]
The research approach used in this study is a cross-sectional design. The study examines the relationship between university-industry collaborations and the creation of university spin-offs at a specific moment in time.	The study used data from the Association of University Technology Managers' (AUTM) Statistics Access for Technology Transfer (STATT) database. The data includes information on the number of spin-offs created by universities, as well as the number of joint patents developed between universities and companies.	The research methods used in this study can be categorized as quantitative research methods.			The study utilized a cross-sectional design, which limits the ability to establish causality between variables or track changes in individual-level outcomes over time. Future research could benefit from adopting panel data methods to overcome this limitation and conduct a more comprehensive analysis of causality. The study focused on data from selected universities, which may limit the generalizability of the findings to other countries or regions. Future research could explore the generalizability of the results in different contexts.	[32]
The text mentions that the study adopted a holistic approach by analyzing simultaneously the extent to which academic staff engage in educational collaboration, research collaboration, and academic entrepreneurship.	Type of data used in this study was a unique primary data collected at 17 Higher Education Institutions in Rwanda	The research method adopted in this study is mixed			This study was focused on the academic perspective and analyses were made at the individual dimension. This scope constitutes its limitation because, considering the contextual embeddedness of UIC process	[88]
The research approach used in this study was an instrument variable approach for university industry linkages and matching methods for public procurement	Type of data used in this study was survey data from the World Bank Enterprise Survey China (2012) from which, have detailed information on small businesses' innovation as well as on their engagement in linkage formation activities and in public procurement	The research method adopted in this study is mixed, were instrument variable approach and matching methods. The instrument variable approach was used to address endogeneity in examining the effect of university industry linkages on firm level innovation, while matching methods were used to estimate the average treatment effect on the treated firms with regard to their innovation performance in relation to public procurement			The research limitations are: innovation measurement relies on subjective statements, data do not allow for meaningful analysis on the moderating effect of regional institutional heterogeneity, and data rely on observable characteristics	[89]
The research approach used in the study was an inductive approach based on the insights offered by Eisenhardt (1989) and Gioia et al. (2013)	The study used a combination of qualitative data obtained through interviews with representatives from technology transfer offices (TTOs), business incubators, investors, and spinoff companies. Additionally, firm records from Bureau van Dijk's FAME database were used to complement the dataset. The data included information on spinoff companies formed between 1959 and 2013 from 87 UK universities.	The research method adopted in this study is mixed and utilized qualitative research methods, specifically interviews, to collect data from representatives of technology transfer offices (TTOs), business incubators, investors, and spinoff companies. A total of 15 interviews were conducted, lasting an average of 40 min, and were voice-recorded or transcribed.			The study acknowledges several limitations. Firstly, the data used, particularly the Spinouts UK data, is not a comprehensive, official, or representative list of spinoff companies, requiring careful handling and cross-validation to ensure accuracy. Secondly, the study focused on exemplary cases of spinoff companies, limiting the understanding of a broader distribution of outcomes. Additionally, the research sample size of 15 interviews may not be sufficient for generalizability, although it complied with guidelines for qualitative research samples. Lastly, the study acknowledges the potential bias in the selection of interviewees and the reliance on self-reported data, which may introduce subjectivity and social desirability bias.	[90]
The research approach used in this article is an exploratory approach, specifically employing an empirical case study analysis.	The study used primary data collected through a survey. The survey included closed questions that captured information about various aspects of university-industry relationships, such as motivations for cooperation, frequency of use of different channels of knowledge transfer, and the importance of specific channels for knowledge transfer.	The research method adopted in this study is mixed and utilized a Two-Sample *t*-Test for Equal Means to examine the significance of differences in motivations for university-industry cooperation between the two groups. The study used descriptive statistics to analyze the degree of importance attributed to different motivations by companies using the Aida database.			The sample used in the study could be expanded to provide more levels of analysis of technological domains, allowing for a deeper exploration of the industry effect on patterns of university-industry cooperation. A larger sample size would enhance the generalizability of the findings. The study did not include a comparative analysis with other regions in different stages of development. Conducting such a study would contribute to a greater extent of validation of the propositions and provide a broader perspective on the patterns of university-industry cooperation.	[91]

Tables 3 and in particular, provides a comprehensive analysis of these articles, categorising them according to data types and research methods. A comprehensive examination of Table 2, Table 3 enriches our insight into the dominant research methodologies, effectively distinguishing between quantitative and qualitative approaches. Furthermore, it delves into the specific data types used to support the research, thereby illuminating the research framework and outlining the limitations inherent in the field of university spin-off creation (USO) through university-industry collaboration (UIC). This methodological approach provides a solid foundation for our study and ensures the quality and reliability of our findings.

## 4 Results

### 4.1 Descriptive characteristics

#### 4.1.1 The most relevant sources applying Bradford's law to reconstruct the landscape of UIC in public USOs creation

**Fig. 2:**
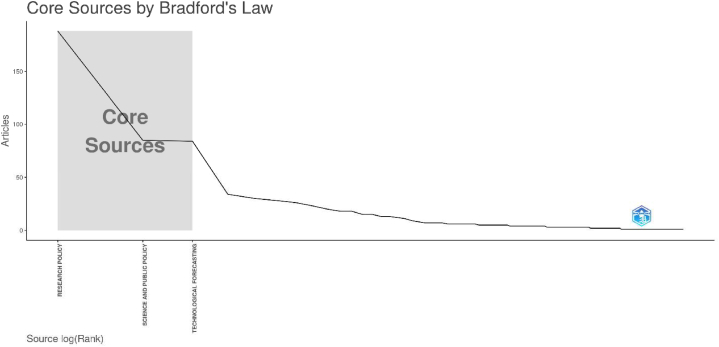
Core sources by Bradfords' Law and the ten most relevant sources identified in our study.

Our analytical focus encompassed a total of 37 carefully selected articles, scattered across a diverse range of 10 different academic journals. Within this scholarly landscape, Research Policy, a prominent British journal publishing original research in the field of “innovation studies”, stood out as the most relevant source, attracting considerable attention, accounting for a remarkable 19% of the total number of articles included in this review and ranking at the top of Zone 1 of Bradfor's Law. It's worth noting that at the time of this research, The Research Policy journal was positioned in the Q1 quartile of quotations and was included in the Social Sciences Citation Index (SSCI) within the Web of Science core collection with an h-index = 61 and a citescore of 15.1 (see Fig. 2).

To gain further insight into the prominence and distribution of publications in the area of UIC in public USOs creation, we used Bradford's Law as a bibliometric lens. This widely recognised tool, as explained by Kör [92], articulates the concept that publications in a given discipline can be categorized into three distinct zones: the core, the middle and the periphery. In the context of our analysis, the core represents a select cluster of journals that contain the preponderance of relevant articles, as illustrated in Fig. 2. In this study, Bradford's Law was concentrated in three Q1 journals as follows: Research Policy, Science and Public Policy, and Technological Forecasting and Social Change.

**Fig. 3:**
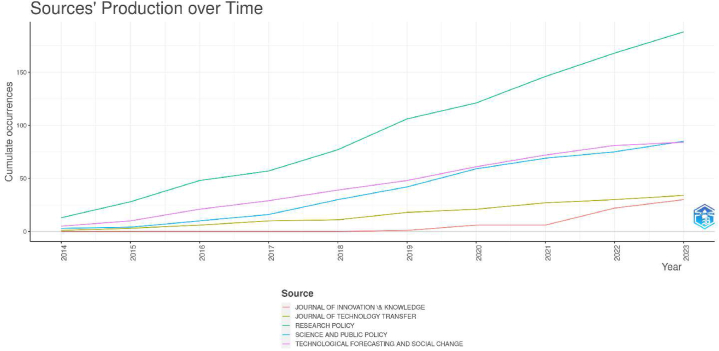
Sources dynamics of production over the period 2014–2023 in the field of UIC in public USOs.

The middle zone includes a set of journals that, while containing a smaller number of relevant articles than the core, still have a significant position of relevance. Finally, the peripheral zone includes a number of journals that contain articles of comparatively lower relevance, as shown visually in Fig. 2, Fig. 3 and Table 4. This stratification, underpinned by Bradford's Law, provides a nuanced lens through which to discern the distribution and significance of scholarly contributions in our chosen field of investigation. The journals considered in this review were carefully curated to closely align with the subject area of public university spin offs (USOs) and University-Industry (U–I) collaboration.

**Table 4:** List of the top ten journals in research on public USOs and UIC.

Sources	Rank	Freq	cumFreq	Zone
Research Policy	1	188	188	Zone 1
Science and Public Policy	2	85	273	Zone 1
Technological Forecasting and Social Change	3	84	357	Zone 1
Journal of Technology Transfer	4	34	391	Zone 2
Journal of Innovation \& Knowledge	5	30	421	Zone 2
Industry and Innovation	6	28	449	Zone 2
Journal of Responsible Innovation	7	26	475	Zone 2
Small Business Economics	8	23	498	Zone 2
Business Strategy and The Environment	9	20	518	Zone 2
Technology Analysis \& Strategic Management	10	18	536	Zone 2

These journals, shown in Fig. 2, Table 4, were predominantly specialist publications, characterised by a strong focus on topics and issues that closely overlapped with our research focus (public USOs and UIC). To ensure the precision of our selection process, we applied rigorous filters that effectively excluded generalist journals, ensuring that the publications under review were finely tuned to the nuances of our research. For instance, prestigious journals such as the Academy of Management Review, the Academy of Management Journal, the Journal of International Financial Management and Accounting, and Management Review, among others, were deliberately omitted from our analysis. This deliberate omission was guided by our desire to focus on journals with a strong thematic emphasis on university-industry collaboration in academic spin-offs, thereby enhancing the relevance and specificity of our review.

As outlined in Table 4, Fig. 3, a remarkable increase in article production in the area of UIC in public USOs was observed in 2019. This upward trend continued in subsequent years, including the challenging period of the COVID-19 pandemic (2020–2022), marking a remarkable recovery. During the 2019–2023 period, the Research Policy Journal emerged as the dominant force in the field of university-industry collaboration in public university spin-offs (U–I collaboration in public USOs), with an impressive total of 188 published articles.

A close second was the Science and Public Policy Journal, with 85 articles contributing significantly to the scholarly discourse. In third place, the Technological Forecasting and Social Change Journal made a significant mark with 84 articles published (see Table 4). This distribution is remarkably consistent with Bradford's Law, as visually depicted in Fig. 2, and highlights the concentration of research output within these three influential journals. It's significant to recognise that all three journals are firmly in zone 1, underlining their pivotal role as primary sources for publications in the field. This insight provides a comprehensive snapshot of the evolving scholarly landscape surrounding UIC in public USOs over the period under consideration, from 2014 to 2023.

#### 4.1.2 Temporal evolution of production (2014–2023) and author origins per country

**Fig. 4:**
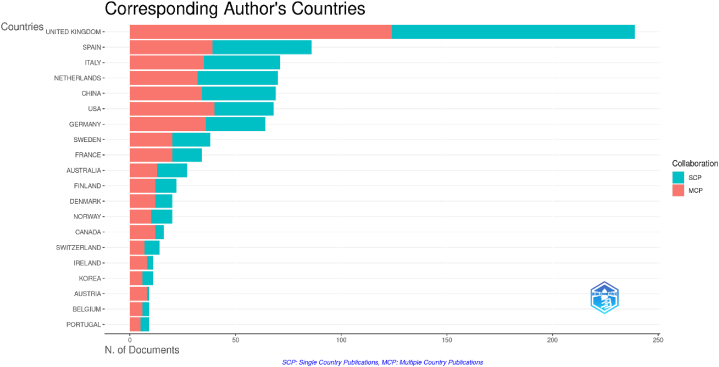
The “three field plot”. A triptych visualisation of UIC in USOs creation: Country of origin of the most relevant authors, prominent authors and keywords-plus identified in our study.

**Fig. 5:**
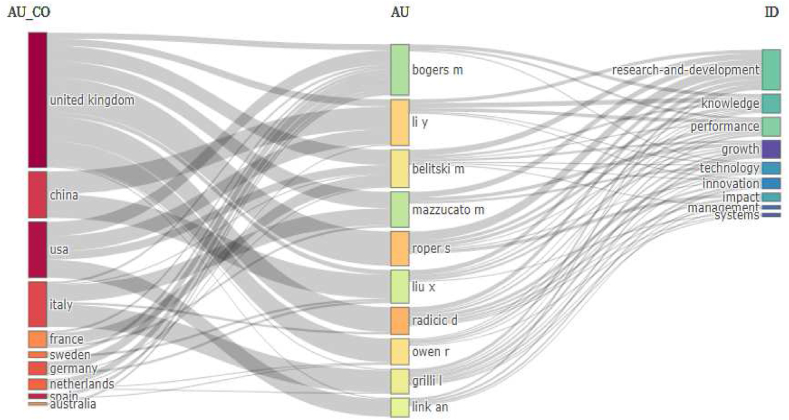
Geographical distribution of corresponding authors in university-industry collaboration within university spin-offs: Single country vs. Multiple country publications.

As we examine the intellectual landscape of this discipline, we highlight the eminent authors whose contributions resonate within this specialised field. These eminent scholars, shown in Fig. 4, are not only identified, but also correlated with their countries of origin and the thematic keywords-plus that characterise their published works. This nuanced perspective offers a comprehensive view of the dynamic interplay between prolific scholars, their research interests and their geographical affiliations (Fig. 5), enriching our understanding of the scholarly tapestry that underpins university-industry collaboration in public USOs.

Fig. 2, Fig. 3, Fig. 4 provide a compelling insight into the global landscape of scientific contributions to university-industry collaboration in academic spin-offs. This analysis highlights the key role played by different countries in this dynamic field and sheds light on both expected and surprising trends. At the pinnacle of this distribution, United Kingdom emerges as a formidable force, asserting its dominance with a substantial 239 publications. Within this impressive score, an intriguing distinction emerges: 124 of these publications take the form of Multiple Country Publications (MCP), reflecting a robust commitment to international collaboration, while the remaining 115 publications fall into the Single Country Publication (SCP) category, highlighting United Kingdom's capacity for independent scholarly output (Fig. 5, Table 5).

**Table 5:** List of the top ten most relevant countries by corresponding author identified in our study.

Country	Articles	SCP	MCP	Freq	MCP_Ratio
United Kingdom	239	115	124	0.239	0.519
Spain	86	47	39	0.086	0.453
Italy	71	36	35	0.071	0.493
Netherlands	70	38	32	0.07	0.457
China	69	35	34	0.069	0.493
USA	68	28	40	0.068	0.588
Germany	64	28	36	0.064	0.563
Sweden	38	18	20	0.038	0.526
France	34	14	20	0.034	0.588
Australia	27	14	13	0.027	0.481

Spain is a distant second with a remarkable 39 MCPs and 47 SCPs. This position underlines Spain's ability to foster both international partnerships and national research efforts. Italy, in third place, shows an interesting dynamic. The contrast is striking: 35 MCP publications reveal a willingness to collaborate across borders, while only 36 SCP publications underline Italy's robust individual research contributions. However, an interesting plot twist emerges when China enters the scene. In fifth place in this global discourse, China's presence is notable, especially as it is in line with the US and the Netherlands. With almost as many contributions published (69), China shows an impressive commitment to international collaboration.

**Fig. 6:**
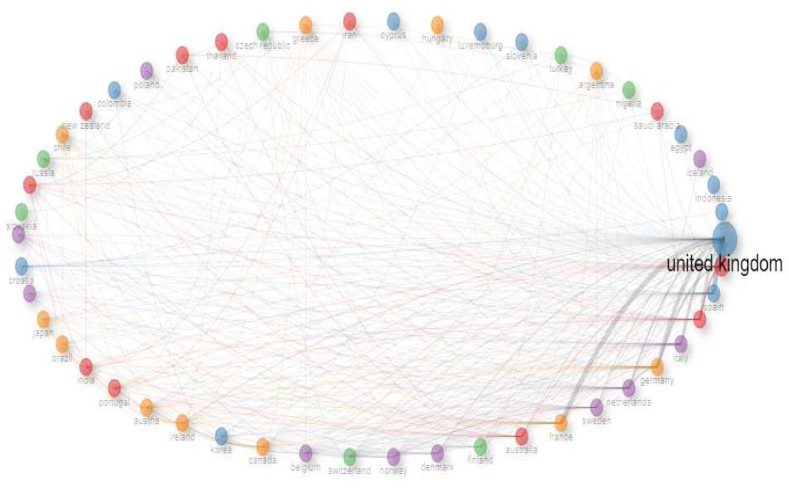
The dynamics of international cooperation in the creation of USOs through UIC.

At the same time, it also produces 35 SCP and 34 MCP publications, reflecting a substantial body of independent research (Table 5). Fig. 6 provides a comprehensive overview of how the scientific landscape of university-industry collaboration in academic spin-offs has evolved over time.

Within the tableau of Fig. 6, the narrative unfolds and it becomes clear that the United Kingdom is reprising its role as the epicentre of collaboration in this domain. Here it is joined by a constellation of collaborators, each representing a unique node in this extensive network of cooperation. Among the key collaborators, we note the active involvement of nations such as Spain, Italy, Germany, Sweden, the Netherlands, France and Australia. Their inclusion underlines the global reach and relevance of these collaborative efforts, and is a testament to the interconnected nature of academic spin-offs and their international reach. Country production over the period considered here, 2014–2023, is shown in Table 6.

**Table 6:** List of top ten of country production over time (2014–2023).

Country	Frequency of articles published 2014–2023
United Kingdom	864
USA	321
Spain	293
China	289
Italy	262
Germany	258
Netherlands	246
Sweden	180
France	147
Australia	115

Fig. 6, in synergy with our previous analyses, paints a vivid picture of the dynamic and interconnected nature of research in university-industry collaboration in academic spin-offs. It underlines the central role of international collaboration in advancing the field and highlights the United Kingdom as an exemplary nation driving global research excellence. Ultimately, this holistic view enriches our appreciation of the complex dynamics and global reach of academic research in this vital field.

#### 4.1.3 The most prominent affiliations where UIC occurs in the creation of public USOs

In the realm of leading research institutions, the University of Utrecht in the Netherlands stands out, contributing a remarkable total of 51 articles to the discourse. It is closely followed by Lund University in Sweden, with a substantial presence of 49 articles, and the University of Manchester in the United Kingdom, with a significant contribution of 44 articles (see Table 7). What is particularly remarkable among the top ten institutions leading the ecosystem of public university spin-offs creation and university-industry collaborations is the notable dominance of the United Kingdom, which boasts six of the top ten universities in this field, contributing a total of 190 research papers on the subject. Surprisingly, Spain, and in particular the Universitat Politècnica de València, a prestigious public university, occupies a commendable fifth place with 33 articles. This comprehensive analysis sheds light on the global landscape of research contributions in the field of spin-offs creation from public universities and their collaboration with industry (Table 7).

**Table 7:** List of the top ten academic institutions contributing the most to research on UIC in public USOs creation.

Affiliation	Country of origin	Number of articles
Utrecht University	Netherlands	51
Lund University	Sweden	49
The University of Manchester	United KIngdom	44
University of Sussex	United KIngdom	41
Universitat Politècnica de València	Spain	33
University of Oxford	United KIngdom	29
Technical University of Munich	Germany	28
Northumbria University	United KIngdom	26
University of Cambridge	United KIngdom	25
University of Warwick	United KIngdom	25

#### 4.1.4 The most relevant authors

**Fig. 7:**
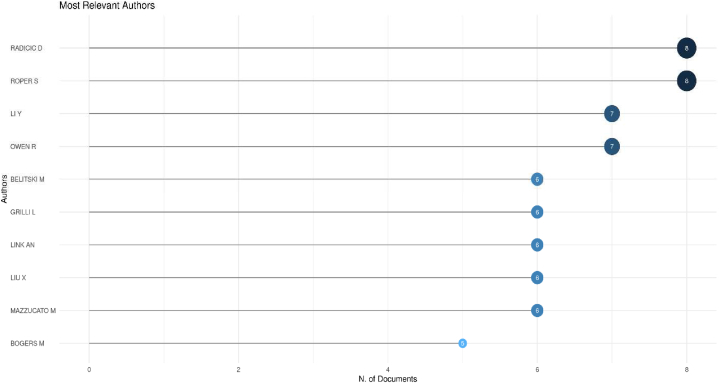
The most relevant authors based on the number of publications on UIC in USOs.

The top ten of the most relevant authors are listed in Table 8 and the most relevant authors based on the number of publications and h-index (cite-factor) are shown in Fig. 9. Through a comprehensive synthesis of the data distilled in Table 8 and Fig. 7, a nuanced understanding emerges. It is evident that within the landscape of our study, Radicid, with an impressive portfolio of 8 articles, began his publication trajectory in 2017 and currently maintains an h-index of 6. Similarly, Roper, with an equivalent amount of 8 articles, began his scholarly contributions in 2014 and matches Radicid's h-index at 6.

**Table 8:** List of the top ten most local cited authors based on the number of publications on UIC in public USOs, 2014–2023.

Element	h_index	g_index	m_index	TC	NP	PY_start
Li Y	7	7	0.778	113	7	2015
Grilli L	6	6	0.6	162	6	2014
Mazzucato M	6	6	0.75	800	6	2016
Radicic D	6	8	0.857	156	8	2017
Roper S	6	8	0.6	424	8	2014
Hughes M	5	5	0.5	199	5	2014
Khan Z	5	5	0.625	185	5	2016
Link An	5	6	0.5	147	6	2014
Parrilli Md	5	5	0.625	212	5	2016
Rogge Ks	5	5	0.625	893	5	2016

Of these notable contributors, Li stands out as a major academic, with seven scientific publications to his name. His scholarly construction began in 2015, and he achieves the distinction of having the highest h-index within this cohort, which stands at an impressive 7. It is particularly important to highlight that Li emerges as an outstanding co-author, actively contributing to the largest corpus of articles in the area of university-industry collaboration in academic spin-offs, as meticulously detailed in Table 8, Fig. 7.

**Fig. 8:**
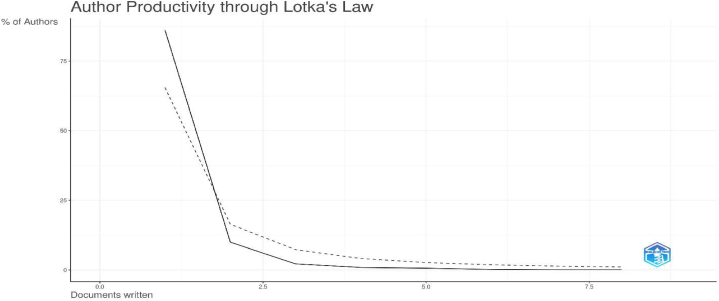
Author productivity on UIC in USOs through Lotka's law.

Table 9 and Fig. 8 both show author activity in the area of UIC on USOs creation through the entire dataset we examined from the Web of Science core collection in the social science index based on Lotka's law. Fig. 8 provides an insightful visualisation of author productivity as assessed by Lotka's Law, a key metric in bibliometric and scientometric analyses. Lotka's Law, also known as Lotka's Frequency Distribution of Scientific Productivity, highlights the intricate distribution of scholarly output within a given field, as elucidated by Qiu et al. [93]. This metric provides a quantitative lens through which to measure the spectrum of production, revealing the exact number of authors responsible for a given number of publications.

**Table 9:** Correlation between authorship and documents on University-Industry collaboration in USOs creation over the period 2014–2023.

Documents written	N. of Authors	Proportion of Authors
1	2094	0.861
2	242	0.099
3	52	0.021
4	21	0.009
5	15	0.006
6	5	0.002
7	2	0.001
8	2	0.001

At its core, Lotka's Law manifests as a power-law distribution, a phenomenon that clearly delineates a select cadre of “prolific authors” who, according to Qiou et al. [93], account for an outsized proportion of scientific contributions. At the same time, it underscores the prominent observation that the broader cohort of authors tends to produce a modest or negligible number of publications (Table 9, Fig. 8).

Within the context of our study, an interesting manifestation of Lotka's law emerges. Specifically, we find that just two authors, Radici and Ropers, have each authored a remarkable eight articles, representing a remarkably small fraction of the total authorship pool, corresponding to a proportion of 0.001. Similarly, two other authors, Li and Owen, have each written seven articles, reflecting this fractional representation of 0.001. This statistical phenomenon underscores the concentration of prolific authorship in our study of university-industry collaboration on public USOs creation (Table 9, Fig. 8).

#### 4.1.5 Trend topics related to UIC on public USOs creation and most cited research literature

**Fig. 9:**
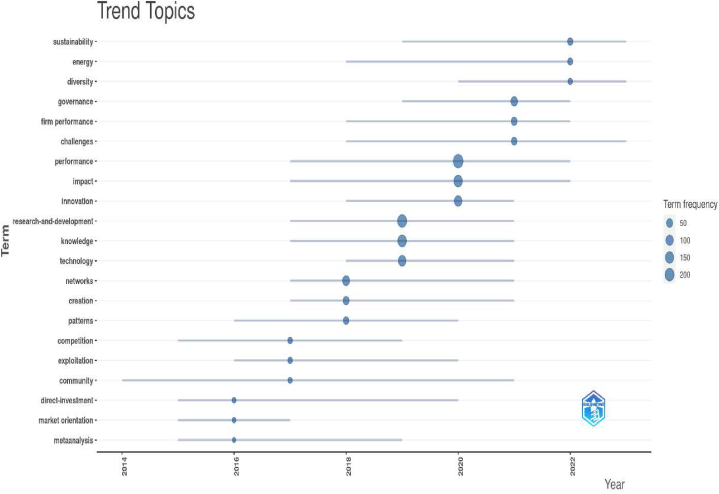
Trend topics ranged from 2014 to 2023 in the field of public USOs creation and UIC.

**Fig. 10:**
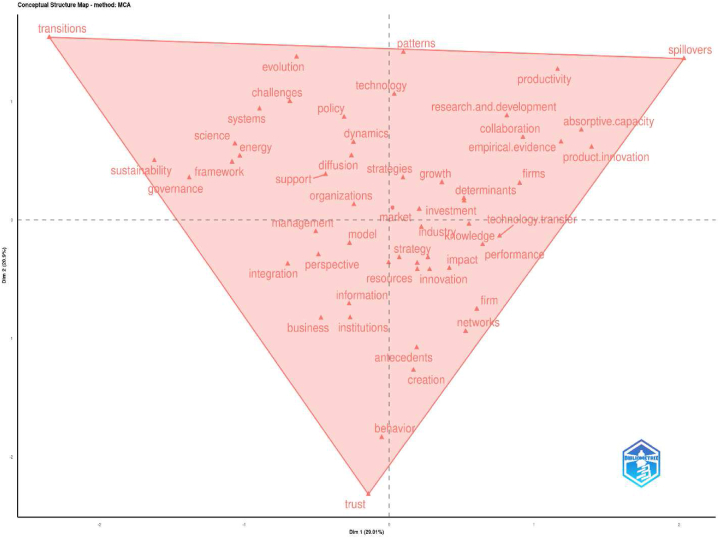
Conceptual structure map based on the MCA method showing transitions, patterns and spillovers of the occurrence of UIC in public USOs creation from 2014 to 2023.

Within the ambit of scholarly exploration, Table 10, Table 11 serve as vital navigational compass points through the intellectual terrain of our comprehensive dataset of 4427 research articles. Derived from the Web of Science Core Collection, specifically the Social Science Citation Index, these tables provide a deep insight into the bedrock of citation prowess. Table 10 reveals with exacting precision the most cited research from our vast repository, each entry representing a beacon of influence and scholarly resonance. These seminal works have, over time, etched their presence in the annals of scholarship, illuminating pathways for subsequent research endeavours. Fig. 9 highlights the different research fronts that reveal both current and potential future trends within the field. In addition, Table 10 presents the thematic map clusters identified in our study and serves as a complementary reference in conjunction with Fig. 9, Fig. 10.

**Table 10:** List of thematic map clusters identified in our study and correlated with the UIC on public USOs creation.

Cluster	Callon Centrality	Callon Density	Rank Centrality	Rank Density	Cluster Frequency
Performance	5.45	10.52	11	1	1738
Management	3.13	12.01	10	5	622
Firm	3.11	15.49	9	8	555
Systems	2.37	10.98	6	3	529
Technology	2.64	11.12	8	4	525
Empirical-evidence	2.37	16.75	7	9	422
Science	1.17	14.22	4	6	250
Sustainability	2.08	10.94	5	2	215
Agency	0.27	18.50	3	11	58
Multilevel perspective	0.21	14.47	2	7	50
Community	0.18	17.36	1	10	42

**Table 11:** List of the ten most cited research papers out of 4427 articles published on public USOs and UIC, 2014–2023.

Paper	DOI	Total Citations	TC per Year	Normalized TC
Schot j, 2018, Res Policy	10.1016/j.respol.2018.08.011	571	95.17	11.00
Colombo mg, 2015, Entrep Theory Pract	10.1111/etap.12118	515	57.22	7.83
Rogge ks, 2016, Res Policy	10.1016/j.respol.2016.04.004	514	64.25	6.79
Kivimaa p, 2016, Res Policy	10.1016/j.respol.2015.09.008	506	63.25	6.68
Teece dj, 2018, Res Policy	10.1016/j.respol.2017.01.015	476	79.33	9.17
Clarysse b, 2014, Res Policy	10.1016/j.respol.2014.04.014	401	40.10	6.27
Coad a, 2016, Res Policy	10.1016/j.respol.2015.10.015	400	50.00	5.28
Stephan u, 2015, J Int Bus Stud	10.1057/jibs.2014.38	372	41.33	5.65
Mazzucato m, 2018, Ind Corp Change	10.1093/icc/dty034	327	54.50	6.30
Felin t, 2014, Res Policy	10.1016/j.respol.2013.09.006	308	30.80	4.81

Meanwhile, Table 11 presents the most cited references within our impressive dataset. These references, valued and recognised in a wide range of scholarly endeavours, are the pillars upon which the edifice of knowledge is built. Their repeated citation underscores their fundamental role in shaping discourse and fueling the intellectual debate that drives our field forward.

Fig. 10 presents a comprehensive conceptual structure map, constructed using the Multicriteria Analysis (MCA) method, that provides insights into the intricate dynamics of the evolution of UIC on public university spin-offs creation over the period 2014–2023. This graphical representation is divided into three distinct components, each of which contributes to a holistic understanding of this collaborative landscape: transitions, patterns, spillovers, and trust. These components are thoughtfully linked to form a triangular framework, anchored at the base by the fundamental concept of “trust”.

At the centre of the map (Fig. 10) are the “patterns” that highlight potential pathways leading to significant spillovers. These patterns include research and development, collaborative initiatives, knowledge, absorption capacity, strategic planning, investment, strategies, the creation of synergistic networks and their cumulative impact on fostering innovation. These patterns serve as guiding principles for steering collaborative efforts towards the outcomes and realisation of spillovers. In the right panel, the map depicts “spillovers”, which encompass the effects of UIC, facilitated by the key factor of “trust”. These spillovers extend to critical facets such as technology transfer, the emergence of innovative firms, the generation of new knowledge, and the creation of extensive networks. These broader impacts go beyond the immediate scope of UIC and dynamically shape the landscape of public USOs (Fig. 10).

#### 4.1.6 The landscape of the co-occurrence keywords

**Fig. 11:**
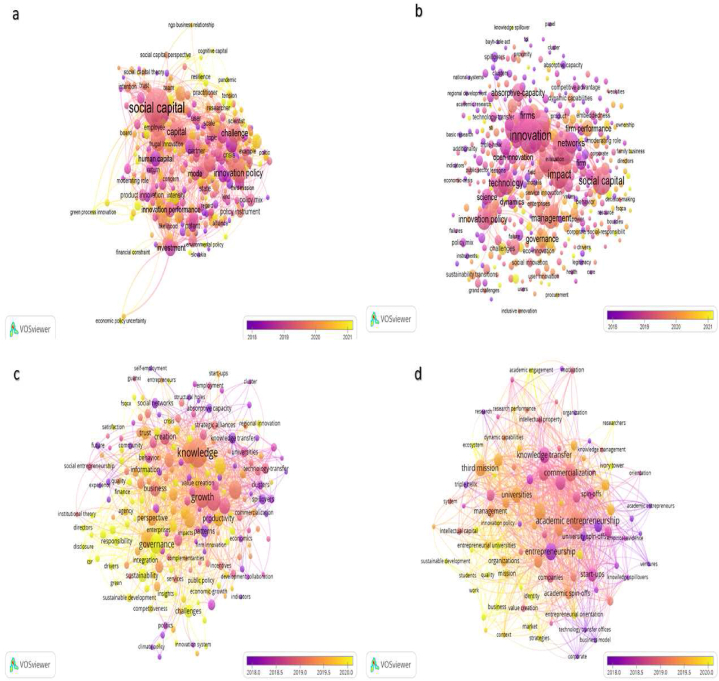
The comprehensive landscape of keyword co-occurrence network on University spin off and UIC. **a)** The co-occurrence network centered on “social capital”; **b)** The co-occurrence network centered on “innovation”; **c)** The co-occurrence network centered on “knowledge transfer”; **d)** The co-occurrence network centered on “third mission of the university”.

Although we analyzed the emerging trends of UIC in public USOs in Fig. 9, Fig. 10 and Table 10, Table 11, Table 12, we still need to show their development over the studied period here, 2014–2023. The timeline map of the co-occurrence keywords can show the occurrence of keywords in different clusters and the time of the first cooccurring relationship among keywords (Fig. 11). We use VosViewer to generate the timeline map based on key word co-occurrence from the reference maneger file. RIS, the time slicing was ranged from 2014 to 2023 with a threshold of 100 keywords from 1844 keywords retrieved from the Web of Science Core Collection data base, specifically from the social science citation index (see Fig. 11).

**Table 12:** List of the ten most cited references included in our study.

Google Scholar	Cited References	Citations
link	Perkmann M, 2013, Res Policy, V42, P423, DOI 10.1016/J.RESPOL.2012.09.007	11
link	D'este P, 2007, Res Policy, V36, P1295, DOI 10.1016/J.RESPOL.2007.05.002	8
link	Cohen WM, 2002, Manage Sci, V48, P1, DOI 10.1287/MNSC.48.1.1.14273	7
link	D'este P, 2011, J Technol Transfer, V36, P316, DOI 10.1007/S10961-010-9153-Z	6
link	Rothaermel FT, 2007, Ind Corp Change, V16, P691, DOI 10.1093/ICC/DTM023	6
link	Ankrah S, 2015, Scand J Manag, V31, P387, DOI 10.1016/J.SCAMAN.2015.02.003	5
link	Perkmann M, 2007, Int J Manag Rev, V9, P259, DOI 10.1111/J.1468–2370.2007.00225.X	5
link	Schartinger D, 2002, Res Policy, V31, P303, DOI 10.1016/S0048-7333(01)00111-1	5
link	Agrawal A, 2002, Manage Sci, V48, P44, DOI 10.1287/MNSC.48.1.44.14279	4
link	Ankrah SN, 2013, Technovation, V33, P50, DOI 10.1016/J.TECHNOVATION.2012.11.001	4

Furthermore, there is no pruning on our network. After generating the co-occurring keyword network, we use the timeline function of overlay visualisation with the time line 2018–2021 in control panel, then, we get the final timeline map containing nodes, links, and a color bar. The nodes on the map represent the keywords, and the color of the links indicates the time when the two nodes co-appear for the first time. Compared with the time bar on the right-side bottom, the closer the connection is to the purple color, the closer the co-occurrence time of keywords is to the present. Eleven clusters were identified (Table 10), listed in hierarchical order: firm; empirical-evidence; systems; performance; sustainability; agency; management; technology; science; community and multilevel perspective.

The clustering results show that “performance” [*n* occurrences = 248; cluster label = 4(performance)] “research and development” [*n* occurrences = 198; cluster label = 4 (performance)] “knowledge transfer” [*n* occurrences = 166; cluster label = 4(performance)] “impact” [*n* occurrences = 158; cluster label = 4(performance)] are the top 4 hotspots. However, it can be inferred from Fig. 11 that “innovation” [*n* occurrences = 109; cluster label = 4(performance)] and “social capital” [*n* occurrences = 14; cluster label = 5(sustainability)] and are the most concerned topics, although the terms “policy” [*n* occurrences = 83; cluster label = 8(technology)] “absorptive capacity” [*n* occurrences = 70; cluster label = 4(performance)] and “firm performance” [*n* occurrences = 42; cluster label = 4(performance)] seems to be around in all the hotspots and emerging trends concerning to “University spin off”, “UIC”55 and “Entrepreneurial Universities” (see Fig. 11, Table 13).

**Table 13:** Indicators –centrality degree, closeness and betweenness– calculated for some of the nodes of the UIC on public USOs network.

Document	Centrality (weighted degree)	Document	Closeness (closeness centrality)	Document	Betweenness (betweenness centrality)
Petersen et al. [94]	250.54	Lehmann et al. [36]	0.002	Borah et al. [66]	0.04
Cunningham & Menter [95]	305.80	Knudsen et al. [79]	0.002	Foray & Woerter [96]	0.03
Llopis et al. [97]	233.69	Fischer et al. [61]	0.002	Vega et al. [71]	0.03
Rodriguez et al. [98]	216.22	Mariani et al. [60]	0.002	Fabiano et al. [99]	0.02
Davies et al. [38]	432.64	Siegel et al. [100]	0.002	Archibugi & Filippetti [43]	0.02

## 5 Discussion

### 5.1 The evolution of scientific productivity

In the annals of scientific progress, the post-2019 era has witnessed a determined and remarkable trajectory of scientific productivity, particularly in this study that covers the period 2014–2023, a trend so solid that it defies the odds and persists even in the face of formidable challenges. As shown in Fig. 3, this upward trajectory is a testament to the indomitable spirit and adaptability of the research endeavour. In particular, it persevered through the turbulent waters of the COVID-19 pandemic and a number of other obstacles to the establishment of a university spin-off through the UIC [4,94,101]. This enduring progression, which has consistently defied the odds, owes much to the collaborative synergy between public universities and industry partners, with particular emphasis on the transformative impact of public university spin-offs [43,100]. It symbolises not only the ability to endure trials, but also the remarkable capacity to maintain an upward trajectory, firmly establishing the scientific arena as a central crucible for nurturing innovation and facilitating the widespread dissemination of knowledge.

Building on the analysis of scientific productivity and its evolution, this study delves into the comprehensive landscape of the keyword co-occurrence network, focusing on university spin-offs and university-industry collaboration. This exploration reveals patterns and synergies among key concepts that highlight the dynamics of collaboration and its fruits. In particular, the network centered on “social capital” is characterized by the prominence of terms such as “innovation policy,” “innovation performance,” “challenge,” and “human capital” (see Fig. 11a). These terms underscore the importance of social capital as a critical facilitator of effective collaboration and innovation.

Conversely, the co-occurrence network centered on “innovation” highlights the interconnectedness of “social capital,” “impact,” “absorptive capacity,” “networks,” “technology,” and “innovation policy” (see Fig. 11b). This arrangement shows how innovation serves as a core around which fundamental concepts for the success of university spin-offs orbit, reflecting a complex network of factors that contribute to innovative performance.

Furthermore, the network centered on “knowledge transfer” highlights the co-occurrence of “knowledge”, “growth” and “governance” (see Fig. 11c), suggesting that effective knowledge transfer is a cornerstone for sustainable growth and governance in the innovation ecosystem. This finding reinforces the notion that knowledge transfer between universities and industry is essential to fully leverage innovation and development capabilities.

Finally, the co-occurrence network focused on the “third mission of the university” sheds light on terms such as “commercialization,” “knowledge transfer,” “academic entrepreneurship,” and “university” (see Fig. 11d). This reflects a growing recognition of the importance of universities not only as centers of teaching and research, but also as active players in the knowledge economy, promoting academic entrepreneurship and the commercialization of innovation.

Taken together, these co-occurrence network analyses provide a detailed view of how different interrelated concepts shape the landscape of university-industry collaboration in the creation of spin-offs, and offer insights into the complex and multifaceted factors that contribute to the success of such ventures in the fields of science and innovation.

### 5.2 The collaborative landscape to stimulate the creation of USOs through UIC

In our analysis, we've uncovered substantial disparities in research productivity between the UK and other countries, notably the United States, Spain, China and Australia (as shown in Table 6). The UK is in the lead with a significant contribution of 37%, closely followed by the United States in 2nd place with 34%, Spain in 3rd place with 33.4% and Australia in 10th place with 13.3%. These results shed light on the dynamic landscape of university-industry collaboration in public university spin-offs [1,102].

The UK emerges as a central player in this global collaboration, as shown in Fig. 6. It has a robust network of affiliations and co-authorships with scholars and institutions around the world [100,96], showcasing its critical role in shaping the field. This emphasis on the UK's role highlights its pivotal position in driving international research efforts in academic spin-offs, a testament to its legacy of research excellence and global engagement. The UK's prominence represents the globalisation of research in university-industry collaboration through spin-offs. This internationalisation is crucial in promoting the exchange of ideas, best practice and the rapid dissemination of knowledge. It underlines the importance of fostering global links to advance our collective understanding of university-industry collaboration and the growth of this critical field [4,11,42,66,101].

### 5.3 Indicators, nodes and collaborative networks

Returning to our research questions, Rq1,2 were typified by the datasets used and the typical methods employed to analyze the data in our comprehensive analysis. Our primary dataset was drawn from the Web of Science Core Collection, a vast and respected source of scientific publications [3,17,28,30,35]. For our network analysis, we leveraged the analytical capabilities of three notable software tools: VosViewer®, Gephi 0.10.1® and Posit PBC™. This strategic combination allowed us to effectively process and visualise the data, providing valuable insights into the collaborative landscape. The focus of our analysis is the collaborative network (Rq1-3), and we've highlighted the top five nodes with the highest indices in Table 13. These indices, including weighted degree, closeness centrality and betweenness centrality, are crucial for navigating the multidimensional research terrain of USO creation through UIC.

Our visual representation in Fig. 11 vividly illustrates the dynamic interplay within the network. Here, connected edges coalesce into cohesive clusters, while nodes exert repulsive forces that push less connected entities towards the periphery. It's important to note that this network is not algorithmically rigid; it allows for manual adjustments, fine-tuning of various attributes, and refinement of the network's structural nuances [49,103].

When these analytical tools are applied, highly connected nodes naturally gravitate towards the core of the network, establishing themselves as central figures in our collaborative network. Conversely, isolated nodes find their place at the boundaries of co-occurrence (Fig. 11). This dynamic representation not only provides insights into structural dynamics, but also underscores the functional importance of each node within our intricate web of scholarly interactions.

### 5.4 Development of public USO and UIC keywords and co-cited reference clusters

An examination of the temporal progression of keywords in the field of public university spin-offs (USOs) and university-industry collaboration (UIC) collaboration provides a compelling narrative, in line with the aims of Rq1. In particular, terms such as “value creation”, “trust” and “business performance” have recently emerged as focal points, appearing in the latter stages of our chronological analysis (see Fig. 10, Fig. 11). This chronicle of keyword evolution underscores the dynamic nature of scholarly inquiry in the field. The journey began with the central concept of ‘social capital’, prominently positioned within the largest cluster, confirming that “innovation” remains the overarching theme in the field of public USOs and U–I collaboration (as illustrated in Fig. 11). This evolution reflects the shifting contours of academic inquiry, illustrating how the field has matured and diversified over time, and providing valuable context for our research and the broader landscape of this interdisciplinary field.

However, it's crucial to distinguish that in the context of public USOs and UIC, “social capital” and its close cousin, “absorptive capacity”, although intertwined, play different roles [36,71,104], They act as instrumental tools or conduits, indirectly facilitating successful collaboration. Social capital and innovation act as bridge-builders, connecting different stakeholders across the value chain, fostering collaboration and creating shared value. As catalysts, they contribute to the development of university spin-offs characterised by robust competition, enduring collaboration and sustainable growth in the thriving business and innovation ecosystem [5,60]. Furthermore, the evolving research landscape in this area has shifted towards concepts such as “knowledge transfer” and the realisation of the “third mission of the university” [52,61,99] and the realisation of the “third mission of the university” [5,94,105]. This shift underlines the transformative journey of public USOs creation and UIC. It represents a profound change in which science is more closely aligned with the broader goals of knowledge dissemination and societal impact, enriching the tapestry of research efforts within this central domain [71,94,99].

### 5.5 Insights for strengthening the entrepreneurial ecosystem in public universities

#### 5.5.1 Theorethical contributions

At the core of our scholarly mission is an unwavering commitment to filling gaps and advancing understanding in the field of public university spin-offs (USOs) creation. We embark on an expedition into uncharted territory within this complex ecosystem. Our research serves as a guidepost, illuminating obscure aspects of USOs and expanding the boundaries of understanding in this complex landscape. In our rigorous inquiry, we traverse the scientific terrain, driven by a commitment to address critical gaps identified in the existing literature. In particular, seminal papers such as Hossinger et al. [3] has highlighted under-researched factors that hinder the venturing process of USOs, such as relationships with parent organisations and regional contexts, as well as factors at the organisational level, namely organisational level, namely entrepreneurship support programmes, industry ties, research orientation and entrepreneurship education. We seize this pivotal moment as an opportunity to provide comprehensive insights and bridge these perceived gaps. More broadly, our study is in line with the scientific discourse initiated by Padilla et al. [52] who advocate highlighting the significance of absorptive capacity and open innovation in the technology transfer process from universities. In strict adherence to their advice, we adopt a knowledge flow approach and systematically unravel the intricate network of factors influencing the performance of USO creation within this dynamic and evolving ecosystem.

#### 5.5.2 Practical insights

Our analysis has uncovered valuable insights that can significantly impact and enrich the entrepreneurial landscape within public universities. These insights come from a comprehensive exploration of hotspots, co-occurrences, keyword development and emerging trends, and provide critical guidance for stakeholders seeking to foster innovation and entrepreneurship within academic institutions.

The identification of hotspots, particularly around “performance”, “Research & Development”, “knowledge transfer” and “impact”, highlights critical areas of focus within the entrepreneurial ecosystem. Leveraging these hotspots implies directing resources and efforts towards enhancing research and development capabilities, facilitating knowledge transfer mechanisms, and assessing the impact of entrepreneurial endeavours [68,106]. Aligning strategic initiatives with these hotspots enables public universities to systematically cultivate a culture of innovation and entrepreneurship. While “performance” remains a dominant hotspot (Fig. 10), our analysis shows that “innovation” and “social capital” are emerging as equally important and widely debated concerns (Fig. 11). Public universities should seize this moment to invest in programmes that foster innovation and strengthen social capital networks. Encouraging cross-disciplinary collaboration, supporting incubators and accelerators, and fostering a culture of knowledge sharing can foster innovation and the development of robust social capital, creating fertile ground for entrepreneurship to flourish. In particular, concepts such as “absorptive capacity” and “firm performance” permeate various hotspots and emerging trends [9,36,51,71,101].

### 5.6 Future research agenda

The pursuit of a comprehensive future research agenda in the field of university spin-off creation (USO) through university-industry collaboration (UIC) collaboration offers promising prospects. An interesting and central avenue for future research is to explore the integration of expert perspectives, primarily through qualitative research in the national contexts of developing countries such as Colombia. In these regions, the landscape of university spin-off creation through university-industry collaboration is still in its embryonic stages, making it particularly fertile ground for research [75,81]. Public universities can use these concepts strategically to enhance their ability to absorb external knowledge, adapt to changing environments and improve overall performance [71,89].

In fact, it is crucial for policymakers in emerging and developing countries to take proactive measures in formulating effective public policies that stimulate collaborative efforts. These policies should extend support to regional development initiatives, foster innovation hubs and make strategic investments in research and development infrastructure [65,69]. Policymakers have a pivotal role to play in addressing the uncertainties that often loom over venture capitalists. Their strategic interventions can encourage universities to actively support spin-off initiatives, while enhancing their credibility in the eyes of external investors through careful research assessment, evaluation and astute management of intellectual property [75,107]. This concerted effort by policymakers can unleash a wave of increased innovation, streamlined technology transfer, robust economic growth, enhanced regional development, and increased social capital and absorptive capacity [36,106].

Future research efforts should delve into a comprehensive exploration of the economic impact generated by the collaborative efforts of universities and industry, and the role they play in shaping the national innovation ecosystem [65]. Furthermore, it is clear that the unwavering pursuit of research intensity and excellence, to the exclusion of other vital functions, has far-reaching consequences for regional development [84].

This points to the need for a systematic application of the ecosystem model to specific academic disciplines, which requires a concerted, well-coordinated effort [90]. It also underlines the importance of implementing mission models within the triple helix framework [79], particularly with regard to the commercialization of publicly funded research results and the key role of intermediaries in facilitating the transfer of research results to the wider societal landscape. It also highlights a future research agenda that emphasises the importance of conducting more in-depth research on specific collaborative initiatives [64,84,107], collecting real-time data and adopting a mixed-methods approach. This multi-faceted strategy is essential to gain a holistic and comprehensive understanding of network formation and the intricate links between different dimensions of social capital within these collaborations [42,78,84].

## 6 Conclusions

In our comprehensive research spanning the decade from 2014 to 2023, it is abundantly clear that the university-industry nexus is a matter of deep concern in academic, managerial and professional circles. This heightened focus underscores the enduring relevance and dynamism of UIC, not only as an academic domain, but also as a pragmatic, real-world imperative. While the United Kingdom stands out as a notable example of robust university-industry engagement, it's important to recognise that European countries, while active participants, still have untapped potential for expanding and strengthening UIC. In contrast, the Asian landscape presents an interesting picture, with China and Japan emerging as prominent proponents of fostering UIC.

UIC has gone beyond being a mere adjunct to research and has become an integral part of meeting the innovation needs of both universities and companies. The undercurrent of this collaboration is undoubtedly technology transfer, which provides a channel for knowledge exchange between these stakeholders. However, as recent publications show, new paradigms have begun to take centre stage. Themes such as “public policy”, “regional development”, “research and development”, “developing countries” and “social capital” have played a central role in shaping the trajectory of UIC. These themes resonate not only with scholars but also with policymakers and serve as guideposts for the next phase of this evolving landscape.

The implications of our findings extend in several directions. Policymakers, especially in developing countries, are urged to adopt a more holistic and proactive stance towards fostering UIC. This includes designing effective public policies that not only facilitate, but also incentivise collaboration. In addition, regional development initiatives that support innovation hubs should be prioritised alongside substantial investment in R&D infrastructure. As the entrepreneurial landscape continues its dynamic evolution, we envision our study becoming an invaluable compass, guiding scholars, policymakers and practitioners alike. It will continue to facilitate exploration and progress in this ever-evolving field, shaping the landscape of university-industry collaboration in public university spin-off creation.

For researchers, our work serves as a beacon, illuminating the changing research terrain within UIC. The emergence of these new themes suggests fertile ground for future investigation. Scholars are encouraged to delve into the intricacies of public policy and analyze its profound impact on UIC. The role of social capital is a rich area for exploration, and examining how developing countries can harness U–I collaboration for sustainable development promises to be a rewarding endeavour. The dynamic nature of this field promises to be a wellspring of opportunities for scholars eager to contribute to the ever-evolving landscape of university-industry collaboration.

## 7 Limitations

While our content analysis benefited from the robust analytical capabilities of the Posit PBC™ tool, formerly known as R Cloud Studio, it is not without its limitations. This scientific mapping approach, while effective in delineating research trends, tends to overlook the intricacies of study design. As a result, future researchers should explore more advanced analytical tools with enhanced capabilities to unlock their full potential for a deeper understanding of the dynamics of academic engagement.

It's noteworthy that our study was conducted in English, and this choice of language is not without implications. While English has indeed become a global lingua franca, scholars from different linguistic backgrounds can use our research approach as a source of inspiration. They may consider replicating this systematic review in their native languages, thus broadening the scope of academic engagement literature analysis. This move towards linguistic diversity promises to bring new insights and perspectives to this dynamic field, ensuring a more comprehensive understanding of academic engagement across cultures and languages.

## Funding

This work was funded by the Unidad Central del Valle del Cauca- UCEVA, through the project entitled “Determination of the Functional Relevance of the Regulatory Framework for Academic Spin-offs, Compared to the Results of SStakeholder Engagement in the Colombian Context’, with the project code PI-1300-50.2-2023-01.

## Data availability statement

We are committed to the transparent and open availability of all data integral to the composition of this paper. The entire dataset is available as a preprint on ScienceOpen Preprints with the following Digital Object Identifier (DOI): 10.14293/PR2199.000573. v1. Researchers, scholars and interested parties are encouraged to explore and use the provided dataset for further analysis and scholarly endeavours. This commitment to data accessibility is consistent with the principles of openness and reproducibility in scientific research, and fosters an environment of collaborative inquiry and the advancement of knowledge. Inquiries should be directed e-mail: aromero@uceva.edu.co.

## CRediT authorship contribution statement

**Alexander Romero-Sánchez:** Writing – review & editing, Writing – original draft, Visualization, Validation, Software, Project administration, Methodology, Investigation, Formal analysis, Data curation, Conceptualization. **Geovanny Perdomo-Charry:** Supervision, Methodology, Investigation, Conceptualization. **Edy Lorena Burbano-Vallejo:** Writing – review & editing, Visualization, Validation, Supervision.

## Declaration of competing interest

The authors declare that they have no known competing financial interests or personal relationships that could have appeared to influence the work reported in this paper.
